# Transcriptomic and proteomic analyses provide insights into the adaptive responses to the combined impact of salinity and alkalinity in *Gymnocypris przewalskii*

**DOI:** 10.1186/s40643-022-00589-1

**Published:** 2022-09-26

**Authors:** Fulei Wei, Jian Liang, Wengen Tian, Luxian Yu, Zhaohui Feng, Qiang Hua

**Affiliations:** 1 State Key Laboratory of Bioreactor Engineering, East China University of Science and Technology, 130 Meilong Road, Shanghai, 200237 People’s Republic of China; 2 State Key Laboratory of Plateau Ecology and Agriculture, Qinghai University, 251 Ningda Road, Xining, 810016 People’s Republic of China; 3 The Rescue and Rehabilitation Center of Naked Carps in Lake Qinghai, 83 Ningzhang Road, Xining, 810016 People’s Republic of China

**Keywords:** *Gymnocypris przewalskii*, Salinity, Alkalinity, DIA/SWATH

## Abstract

*Gymnocypris przewalskii* is the only high-land endemic teleost living in Qinghai Lake, the largest saline–alkaline lake in China. Its osmoregulatory physiology remains elusive due to a lack of precise identification of the response proteins. In the present study, DIA/SWATH was used to identify differentially expressed proteins (DEPs) under alkaline (pH = 10.1, carbonate buffer), saline (12‰, sodium chloride), and saline–alkaline [carbonate buffer (pH = 10.1) plus 11‰ sodium chloride] stresses. A total of 66,056 unique peptides representing 7,150 proteins and 230 DEPs [the false discovery rate (FDR) ≤ 0.05, fold change (FC) ≥ 1.5] were identified under different stresses. Comparative analyses of the proteome and transcriptome indicated that over 86% of DEPs did not show consistent trends with mRNA. In addition to consistent enrichment results under different stresses, the specific DEPs involved in saline–alkaline adaptation were primarily enriched in functions of homeostasis, hormone synthesis and reactions of defense response, complement activation and reproductive development. Meanwhile, a protein–protein interaction (PPI) network analysis of these specific DEPs indicated that the hub genes were ITGAX, MMP9, C3, F2, CD74, BTK, ANXA1, NCKAP1L, and CASP8. This study accurately isolated the genes that respond to stress, and the results could be helpful for understanding the physiological regulation mechanisms regarding salinity, alkalinity, and salinity–alkalinity interactions.

**Graphical Abstract:**

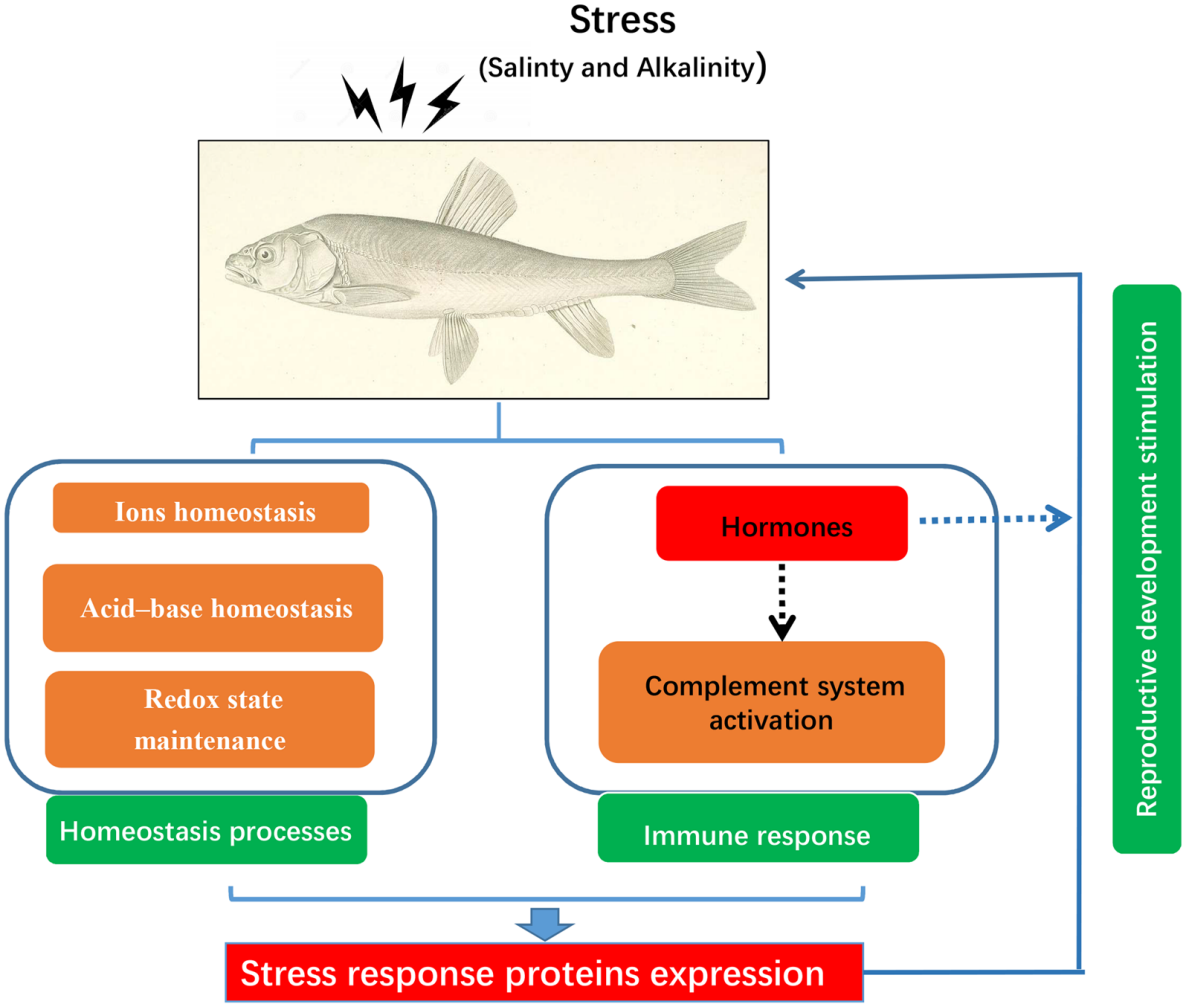

**Supplementary Information:**

The online version contains supplementary material available at 10.1186/s40643-022-00589-1.

## Highlights

1. Effects of salinity, alkalinity and their combined impact were analysed with transcriptome and and proteomics methods in* G*.* przewalskii*.
2. The validity of transcriptome and proteome results in * G*.* przewalskii* was compared.
3. Hub genes in the adaptive responses to the combined impact of salinity and alkalinity were isolated.
4. Physiological regulation mechanisms regarding salinity, alkalinity, and salinity-alkalinity interactions were discussed.

**Supplementary Information:**

The online version contains supplementary material available at 10.1186/s40643-022-00589-1.

## Introduction

Naked carp (*Gymnocypris przewalskii*) is the only endemic teleost in Lake Qinghai, the largest inland lake in China on the Qinghai-Tibet plateau (Yang et al. 2005; Walker et al. 1995). The naked carp has always played an important role in the maintenance of the ecological balance of Lake Qinghai. Approximately 130,000 years ago, due to geological movement and a drought environment, the surrounding mountains of Qinghai Lake were strongly uplifted and the Qinghai Lake became an inland saltwater lake (Yuan et al. 1990). With the gradual increase of salinity and alkalinity of the lake water, the genetic characteristics of *G. przewalskii* have gradually evolved from those of a freshwater fish to a species that is adapted to the saline and alkaline environment of the plateau. In contrast to lowland teleost fish species, an elevated genome-wide rate of molecular evolution in *G. przewalskii* has been observed. A set of rapidly evolving ion transport genes and transcriptomic signatures in this Schizothoracine fish associated with adaptation to the saline and alkaline environment of the Tibetan plateau were identified using comparative genomics (Tong and Li 2020a). Such adaptive changes have also been observed in hybrid tilapia when adapting to saline–alkaline conditions (Su et al. 2020). Although such studies have examined osmoregulatory physiology in fishes, the molecular mechanism of osmotic stress tolerance remains unclear (Hu et al. 2015; Tong et al. 2017; Tong and Li 2020b; Xu et al. 2013a; Yao et al. 2012; Zhao et al. 2021). This is possibly due to the lack of precise information concerning response proteins and a platform with which to verify their functions.

Previous commercial fishing has caused a sharp decrease of *G. przewalskii* stocks from the 1950s to the 1990s*.* In the predictable future, the naked carp might face critical challenges from the increasing salinity levels of the lake (Shi et al. 2000.). In the last several decades, release and artificial propagation have been used to augment the wild stock. From June to September, gametes of natural mature individuals are collected for artificial insemination when the naked carps migrate into rivers around Qinghai Lake for spawning. After one year of artificial breeding in fresh water ponds in Xining, the 1^+^ year-old fishes are released into Qinghai Lake for artificial propagation. This method can improve the fertilization rate and the survival rate of juvenile fish, however, the severity of the challenge to juvenile naked carp posed by the saline and alkaline lake water is still unknown. In our lab, the naked carps under 7 days of 12‰ salinity and pH 10.1 alkalinity stress exhibited severely hypoxia phenotype and high mortality of 38 ± 8.0% (Additional file 1: Table S1). Thus, we believed the combined stress of salinity and alkalinity imposed challenge to these juvenile individuals. This fact prompted us to examine the saline–alkaline response of the naked carp and the study will be of great significance for guiding the conservation of this species.

With recent advances in the sensitivity of mass spectrometry-based proteomics, it is possible to investigate gene and protein expression profiles using multi-omics approaches. Therefore, we hypothesized that the response to saline and alkaline conditions could be elucidated by analyzing protein expression and comparing the transcriptional and proteomic data.

In the present study, we performed a DIA analysis of the gills of 1^+^ year-old *G. przewalskii* after acute stress. The method combined the advantages of DDA (data-dependent acquisition) and SWATH (sequential window acquisition of all theoretical fragment ions) to examine the physiology of saline–alkaline adaptation in the naked carp. As a result, the main functional genes and signal regulatory pathways were explored. Meanwhile, our data identified the osmoregulation mechanism of the naked carp to saline–alkaline challenge and provided an overview of stimulated pathways and gene expression patterns. Most importantly, the study isolated the genes that were activated in response to stress. The present results will be helpful for understanding the physiological regulation mechanisms of response to salinity, alkalinity, and saline–alkaline interactions as well as guiding conservation efforts for *G. przewalskii.*

## Methods

### Animals

The naked carps provided by The Rescue and Rehabilitation Center of Naked Carps in Lake Qinghai (Xining, China) were juvenile fishes of 1^+^ years of age, 7.5 ± 1.4 cm in length and 4.6 ± 0.9 g in weight. Since the naked carps were far from maturation and it was difficult to distinguish the sex of fish at the juvenile stage, gender factor was not considered in this experiment. After three days of acclimation in fresh water in the laboratory, the naked carps were divided into an alkaline group (pH = 10.1, carbonate buffer) (A), a saline group (12‰, sodium chloride) (S), a saline–alkaline group [carbonate buffer (pH 10.1) plus 11‰ sodium chloride] (SA) and a fresh water group (F) for stress treatment based on pre-experimental results (Additional file 1: Table S1). To prevent death from acute stresses, these final stress intensities were achieved by gradual increment over two days. After 7 days of stress, the fish were euthanized with a lethal dose (80.0 mg/L) of MS-222 (Sigma, Shanghai, China). The gills were dissected from each fish and then frozen in liquid nitrogen until transcriptomic and proteomics experiments were undertaken.

### RNA sequencing and bioinformatic analysis

Total RNA was extracted using a Trizol reagent kit (Invitrogen, Carlsbad, CA, USA). After being assessed on an Agilent 2100 Bioanalyzer (Agilent Technologies, Palo Alto, CA, USA) and by agarose gel electrophoresis, the total RNA was extracted by Oligo (dT) beads in order to enrich the mRNA (Epicentre, Madison, WI, USA). The enriched mRNA was fragmented using fragmentation buffer and reverse transcribed into cDNA using random primers. Following the second-strand cDNA synthesis by DNA polymerase I, the cDNA fragments were purified using a QiaQuick PCR extraction kit (Qiagen, Venlo, The Netherlands). After a poly (A) tail was added, the resulting fragments were ligated to Illumina sequencing adapters. The ligation products with size approximately 200 bp were isolated and PCR amplified, followed by sequencing using an Illumina HiSeq 2500 by Gene Denovo Biotechnology Co. (Guangzhou, China). Reads obtained from the sequencing were filtered by fastp (version 0.18.0) in order to remove reads containing adapters, more than 10% of unknown nucleotides (N), or 50% low-quality (*q-value* ≤ 20) bases (Chen et al. 2018). Transcriptome de novo assembly was carried out with the short reads assembling program Trinity (Haas et al. 2013). To annotate the unigenes, BLASTx with e-value < 0.00001 (http://www.ncbi.nlm.nih.gov/BLAST/) was used to align sequences with the NCBI non-redundant protein (Nr) database (http://www.ncbi.nlm.nih.gov), the Swiss-Prot protein database (http://www.expasy.ch/sprot), the Kyoto Encyclopedia of Genes and Genomes (KEGG) database (http://www.genome.jp/kegg), and the COG/KOG database (http://www.ncbi.nlm.nih.gov/COG). For differentially expressed genes (DEGs) isolation, FPKM (fragment per kilobase of transcript per million mapped reads) value was calculated to quantify its expression abundance and variations using StringTie v1.3.1(Ali et al. 2008). RNA differential expression analysis between different groups was performed by DESeq2 (Love et al. 2014) software and edgeR (Robinson et al. 2010) for comparisons between two samples. The genes/transcripts with FDR ≤ 0.5 and FC ≥ 2 were considered DEGs.

### Protein preparation

The gills were homogenized with lysis buffer (2% SDS, 7 M urea, 1 mg/ml protease inhibitor cocktail) for 3 min on ice using an ultrasonic homogenizer, and the supernatant was collected after centrifugation at 15,000 rpm for 15 min at 4 °C. After protein concentration determination using a BCA protein assay kit (Promega, Madison, WI), the supernatant was diluted to 1 μg/μL and incubated at 55 °C for 1 h with 0.02 M dithiothreitol (Promega, Madison, WI). Then, 5 μl of 1 M iodoacetamide (Promega, Madison, WI) was added to 50 μl diluted supernatant to precipitate the proteins, which were then hydrolyzed with trypsin (Promega, Madison, WI) at a substrate/enzyme ratio of 50:1 (w/w) at 37 °C for 16 h after precipitation with 300 μl of precooled acetone at − 20 °C overnight and redissolution in 50 mM ammonium bicarbonate (Promega, Madison, WI).

### Establishment of a spectrogram database

The peptide mixture was re-dissolved in 20 mM ammonium formate (pH 10.0, adjusted with ammonium hydroxide) (Promega, Madison, WI) and then fractionated by high pH separation using an Ultimate 3000 system (ThermoFisher Scientific, Waltham, MA, USA) connected to a reverse phase column (XBridge C18 column, 4.6 mm × 250 mm, 5 μm) (Waters Corporation, MA, USA). The column flow rate was maintained at 1 mL/min at a temperature of 30 °C. After re-equilibration in 5% buffer A for 15 min (buffer A: 20 mM ammonium formate in 80% acetonitrile, pH 10.0, adjusted with ammonium hydroxide), high pH separation was performed in a linear gradient way, starting from 5% buffer A to 45% in 40 min. Ten fractions were collected, and each was dried in a vacuum concentrator for DDA: nano-HPLC–MS/MS analysis.

In the DDA: nano-HPLC–MS/MS analysis, 30 μL aqueous solution of 0.1% formic acid was used to re-dissolve the dried peptides. Then, 3 μL peptide samples were analyzed using an online nanospray LC–MS/MS on an Orbitrap Fusion Lumos coupled to an EASY-nLC 1200 system (Thermo Fisher Scientific, MA, USA) with an analytical column (Acclaim PepMap C18, 75 μm × 25 cm). The column flow rate was maintained at 200 nL/min at a temperature of 40 °C. The electrospray voltage of 2 kV versus the inlet of the mass spectrometer was used. The analysis was run in data-dependent acquisition mode that was switched between MS and MS/MS modes. The parameters for the MS were as follows: scan range (m/z) = 350–1200; resolution = 120,000; AGC target = 400,000; maximum injection time = 50 ms; Filter Dynamic Exclusion: exclusion duration = 30 s; (2) HCD-MS/MS: resolution = 15,000; AGC target = 50,000; maximum injection time = 35 ms; collision energy = 32.

### Database search

Spectronaut X (Biognosys AG, Switzerland) with default parameters was used to analyze the raw data from the DDA. Once the initial target list was generated, the Spectronaut program was employed to search the database of *G. przewalskii*’s transcriptome that contained 37,684 proteins (the reported sequences data were archived in the Sequence Read Archive (SRA) with accession number PRJNA833655) along with a contaminant database assuming trypsin as the digestion enzyme. Carbamidomethyl (C) was specified as the fixed modification. Oxidation (M) was specified as the variable modification. The *q-value* cutoff on precursor and protein levels was applied as 1% for precursor and protein levels.

### DIA: nano-HPLC–MS/MS analysis and data analysis

For DIA: nano-HPLC–MS/MS analysis, the equipment and experimental process were similar to those for the DDA: nano-HPLC–MS/MS analysis. The parameters were: (1) MS: scan range (m/z) = 350–1200; resolution = 120,000; AGC target = 1e6; maximum injection time = 50 ms; (2) HCD-MS/MS: resolution = 30,000; AGC target = 1e6; collision energy = 32; stepped CE = 5%. (3) DIA was performed with a variable isolation window; each window overlapped 1 m/z, and the window number was 60. Raw DIA data were processed and analyzed by Spectronaut X with default parameters. Retention time prediction type was set to dynamic iRT. Data extraction was determined by Spectronaut X based on the extensive mass calibration. Spectronaut Pulsar X determines the ideal extraction window dynamically depending on iRT calibration and gradient stability. The *q-value* cutoff on precursor and protein levels was applied as 1%. Decoy generation was set to “mutated”, which is similar to “scrambled”, but only applies a random number of AA position swaps (min = 2, max = length/2). All selected precursors passing the filters were used for quantification. The average top three filtered peptides that passed the 1% *q-value* cutoff were used to calculate the major group quantities. After Student’s t-test with R package models (http://www.r-project.org/), DEPs were filtered if their *q-value* was ≤ 0.05 and FC ≥ 1.5. For the protein qualitative analyses, the quality control reagent IRT (Biognosys) was added to each sample before mass spectrometry detection, and the calibration was carried out according to retention time (RT) of polypeptides in chromatography. After quality control with Quic (Biognosys), Pulsar was used to build a database of data obtained from DDA collection mode, and the DIA results were analyzed according to the DDA reference database to identify proteins (Kim, et al. 2018).

In the bioinformatic analysis, the proteins with *q-value* ≤ 0.05 and FC ≥ 1.5 were considered as DEPs. Principal component analysis (PCA) was performed with the R package models (http://www.r-project.org/) in this experiment. To determine the main biological functions of the differentially expressed genes, Gene Ontology (GO, http://www.geneontology.org/) terms were assigned by using Blast2GO (version 2.8) to search the Nr database (r20200419) (Conesa et al. 2005). GO classification was performed using the OmicShare tools, a free online platform for data analysis (http://www.omicshare.com/tools), and the GO categorization results were expressed as 3 independent hierarchies for molecular function, biological process and cellular component. The KEGG pathway annotation was performed using Blastp (version 2.6.0) software against the Kyoto Encyclopedia of Genes and Genomes (KEGG) database (version 3.0) according to the method described by Kanehisa et al. (Kanehisa and Goto 2000). For GO enrichment analysis and KEGG enrichment analysis, all *p-values* were adjusted with the Benjamini and Hochberg (BH) correction (Benjamini and Hochberg 1995) and a corrected *p-value* ≤ 0.05 was chosen as the threshold value to determine significantly enriched GO terms or enriched pathway. Background genelists in that analysis were supplied in Additional files 2 and 3. In addition, A protein–protein interaction network was constructed using String v10 (Szklarczyk et al. 2015), and the network file was visualized using Cytoscape (v3.7.1) (Shannon et al. 2003).

### Western blotting

Crude protein was extracted from 1 g of gill tissue with cell lysis buffer (Beyotime, Shanghai, China). After concentration determination with a BCA protein assay kit (Promega, Madison, WI), protein expression patterns were measured by Western blotting. Quantification was accomplished with relative antibodies as follows: (a) 20 μg of crude protein was separated using SDS-PAGE and then transferred to a PVDF membrane (Merck, USA). (b) The membranes were blocked in TBST buffer (Solarbio, Beijing, China) with 5% skim milk (Solarbio, Beijing, China) for one hour at room temperature. (c) The blocked membranes were incubated with diluted primary antibody (1:1000) for 12 h at 4 °C. (d) The membranes were conjugated with anti-rabbit immunoglobulin horseradish peroxidase-conjugated secondary antibody at a dilution rate of 1:5000 for one hour. (d) Proteins were visualized with ECL detection reagent (Beyotime, Shanghai, China). The primary antibodies for ANXA1, NDRG1, BAX, MMP9 (Art. No: WL0040; WL03071; WL02635; WL03096) and HRP-conjugated Goat Anti-Rabbit IgG (Art. No: WLA023) were from WanleiBio (Shenyang, China). Primary antibodies of β-actin was from Boster (Art. No: BM5422, Wuhan, China) and corresponding HRP-labeled secondary antibodies were from ZSGB-BIO (Art. No: ZB2305, Beijing, China).

### Statistical analysis

The relative expression amount was calculated as the multiple in contrast to freshwater group, origin pro 2010 (OriginLab, USA) was used to analyze and plot the data. Data were expressed as means ± SE (standard error of mean) of three replicates.

## Results

### Proteomic profiling under different stresses

**Fig. 1:**
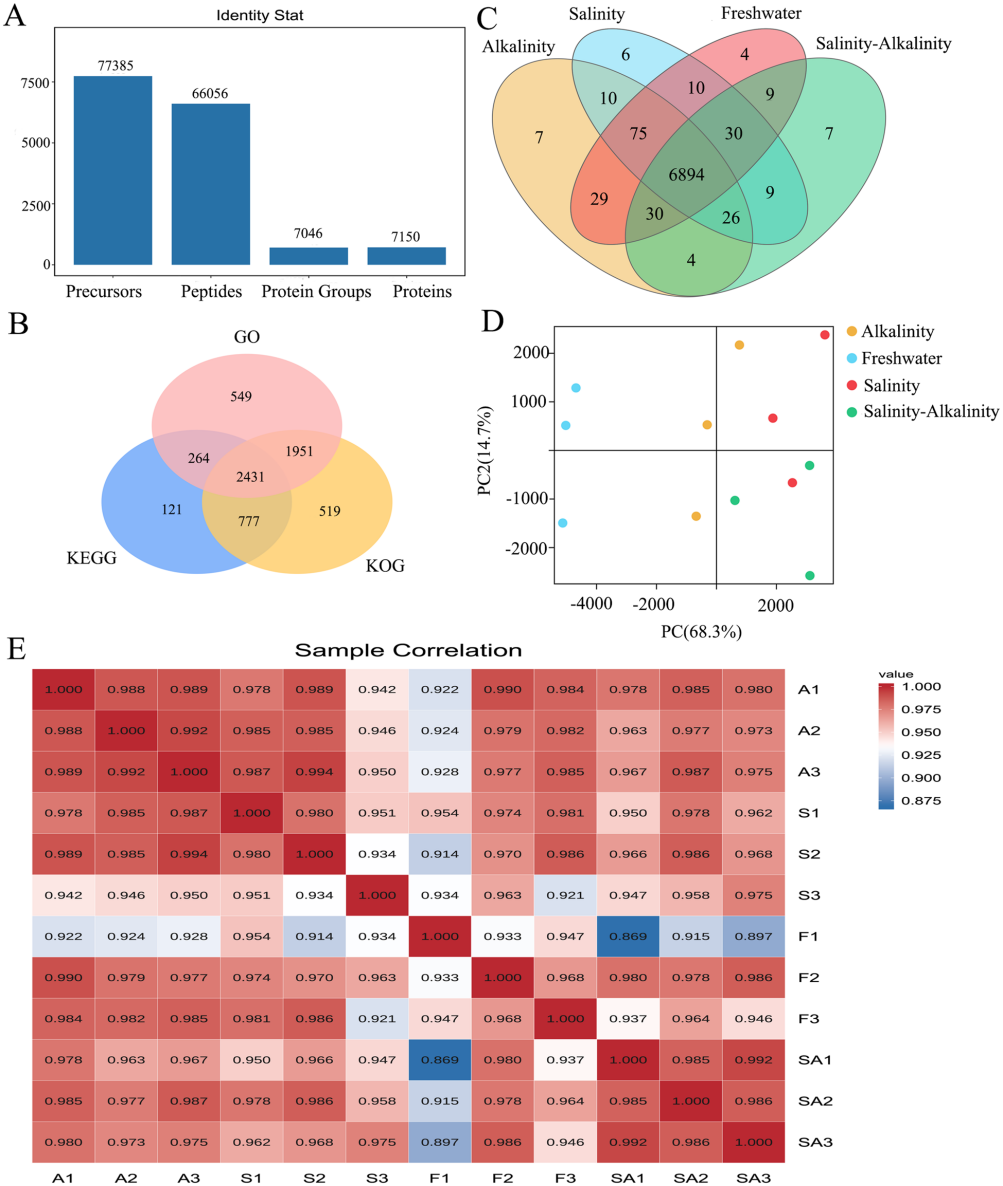
Protein identification and sample relationship analysis. **A** The statistics of protein and peptide identification; **B** Venn diagram of overlap of the proteins with annotation in the GO, KEGG, or KOG databases; **C** number and Venn diagram of overlap of Salinity, Alkalinity, Freshwater and Salinity–Alkalinity groups; **D** principal components analysis (PCA) of different individuals. **E** Correlation analysis of replicates among different groups. S indicates replicate in Salinity stress, A for Alkalinity, **F** for Freshwater and SA for Salinity–Alkalinity

We used the DIA-MS based proteomics approach to quantify the proteome. The data were filtered with the standards of 1.0% FDR for the precursor threshold and protein threshold at the peptide level and protein levels in the qualitative analysis. After merging the filtered data from the four groups, a total of 66,056 unique peptides representing 7,150 proteins were identified (Fig. 1A). On average, one protein was covered by 9.23 peptides. Among the 7,150 proteins, 6,612 had annotations in the GO, KEGG and KOG databases (Fig. 1B). There were 7,081, 7,075, 7,060, and 7,009 proteins separately identified in the Freshwater, Alkaline, Saline, and Saline–alkaline groups. Meanwhile, 6,894 proteins were shared across the four groups (Fig. 1C). Principal component analysis (PCA) (Fig. 1D) and Pearson’s correlation coefficients of protein intensities (Fig. 1E) indicated a good repeatability among the replicated samples within the groups.

### Comparative analysis of the proteome and transcriptome

Here, we used RNA-Seq to establish a database to ascertain the results from the raw data of DDA and to make general comparisons across RNA-Seq and proteomics platforms. After filtering the reads with adapters, reads containing more than 10% N, all A-base reads, and low-quality reads (the base number of Q ≤ 20 accounting for more than 50%), 37.9 to 44.3 million clean reads assembled a total of 79,152 unigenes with a maximum length of 27,026 and a minimum of 201 bases (Additional file 1: Figure S1A). By pairwise comparisons, we found specific transcriptomic changes in response to different various acute stressors. The two most significant differences between groups were Freshwater-vs-Saline (1438) and Freshwater-vs-Saline–alkaline (1513), while the least was Freshwater-vs-alkaline (378) (*q-value* ≤ 0.05 and FC ≥ 2) (Additional file 1: Figure S1B, S1C and S1D). In the KOG function classification, these unigenes were classified into 25 functional categories in which the two largest of the 25 categories were ‘signal transduction mechanism’ (4549) and ‘general function prediction only’ (4578) (Additional file 1: Figure S1E).

**Fig. 2:**
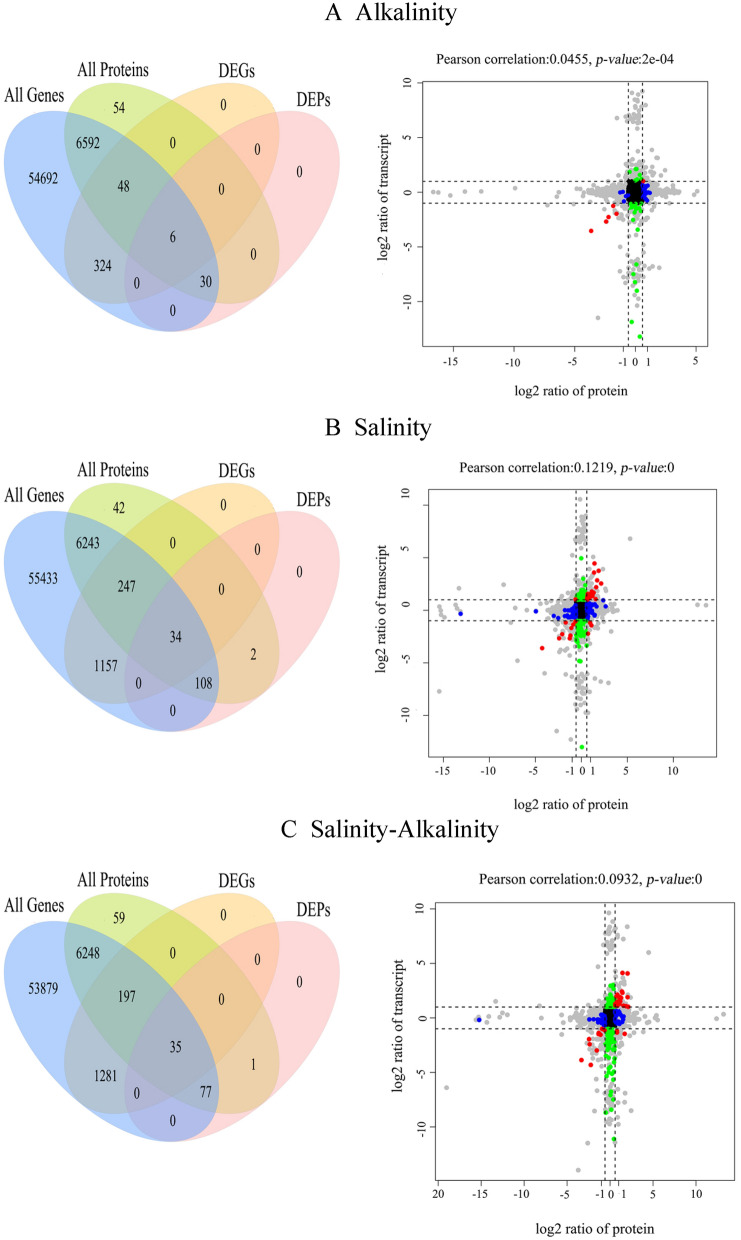
Overlap and the correlation between transcriptome and proteome under Alkalinity (A), Salinity (B) and Salinity–Alkalinity stresses (C). Venn diagrams indicate overlap and quantities of detected genes, proteins, differential genes (DEGs) (the fold change (FC) ≥ 2.0, the false discovery rate (FDR) ≤ 0.05) and proteins (DEPs) (fold change ≥ 1.5, FDR ≤ 0.05). Dotted line in nine-quadrant diagrams means the fold change threshold for the DEGs and DEPs. Each dot represents a gene/protein. Black dots represent non-differential proteins and genes; red dots represent DEGs and DEPs showing consistent or opposite change trends; green dots represent genes differentially expressed but proteins none differentially expressed; blue dots represent genes none differentially expressed but proteins differentially expressed; gray dots represent genes (FC ≥ 2, FDR > 0.05) or proteins (FC ≥ 1.5, FDR > 0.05)

In quantitative analysis of the correlation between transcriptome and proteome, the threshold criteria for DEGs were set as *q-value* ≤ 0.05 and FC ≥ 2, and the threshold criteria of DEPs were *q-value* ≤ 0.05 and FC ≥ 1.5. As shown in Fig. 2, the number of genes identified by transcriptome and proteome association was 6676 for Alkaline, 6632 for Saline, and 6557 for Saline–alkaline. As presented in nine quadrant diagrams, the Pearson's correlation coefficients between protein and transcript were 0.0455 for Alkaline, 0.1219 for Saline and 0.093 for Saline–alkaline, indicating a difference in the trend of transcriptional expression compared to protein abundance (Fig. 2A, B, C). In these diagrams, the filtered DEGs/DEPs (represented with red, green and blue dots in Fig. 2) accounted for 314 in the saline group, 62 in the alkaline group, and 236 in the saline–alkaline group. These DEGs/DEPs can be divided into 3 groups based on the correlations between protein and transcript abundance. (I) The positive correlation subgroup (PC) in which the mRNA level was positively correlated with protein abundance. (II) rNC subgroup indicating a subgroup with high transcripts abundance in which proteins show negative or non-correlation. (III) pNC subgroup representing a subgroup with accumulated proteins in which genes show negative or non-correlation. As shown in Table 1, in these genes, the PC subgroup accounted for only 9.55 to 13.56% in different groups. In contrast, rNC and pNC subgroups in which the mRNA level was not positively correlated with protein abundance accounted for more than 86% under different stresses. This phenomenon indicated that the transcriptome could not completely explain physiological mechanisms of environmental adaptation in naked carp.

**Table 1:** Quantitative analysis of DEGs (*q-value* ≤ 0.05 and FC ≥ 2) and DEPs (*q-value* ≤ 0.05 and FC ≥ 1.5) in transcriptome and proteome correlation under stresses. rNC subgroup indicating a subgroup with high transcripts abundance in which proteins show negative or non-correlation. pNC subgroup representing a subgroup with accumulated proteins in which genes show negative or non-correlation. PC symbolizes a subgroup in which the mRNA level was positively correlated with protein abundance

	rNC	pNC	PC
Salinity	173 (55.06%)	111 (35.35%)	30 (9.55%)
Alkalinity	12 (19.35%)	44 (70.96%)	6 (9.68%)
Salinity–alkalinity	108 (45.76%)	96 (40.68%)	32 (13.56%)

### Pairwise differential abundance analysis

**Fig. 3:**
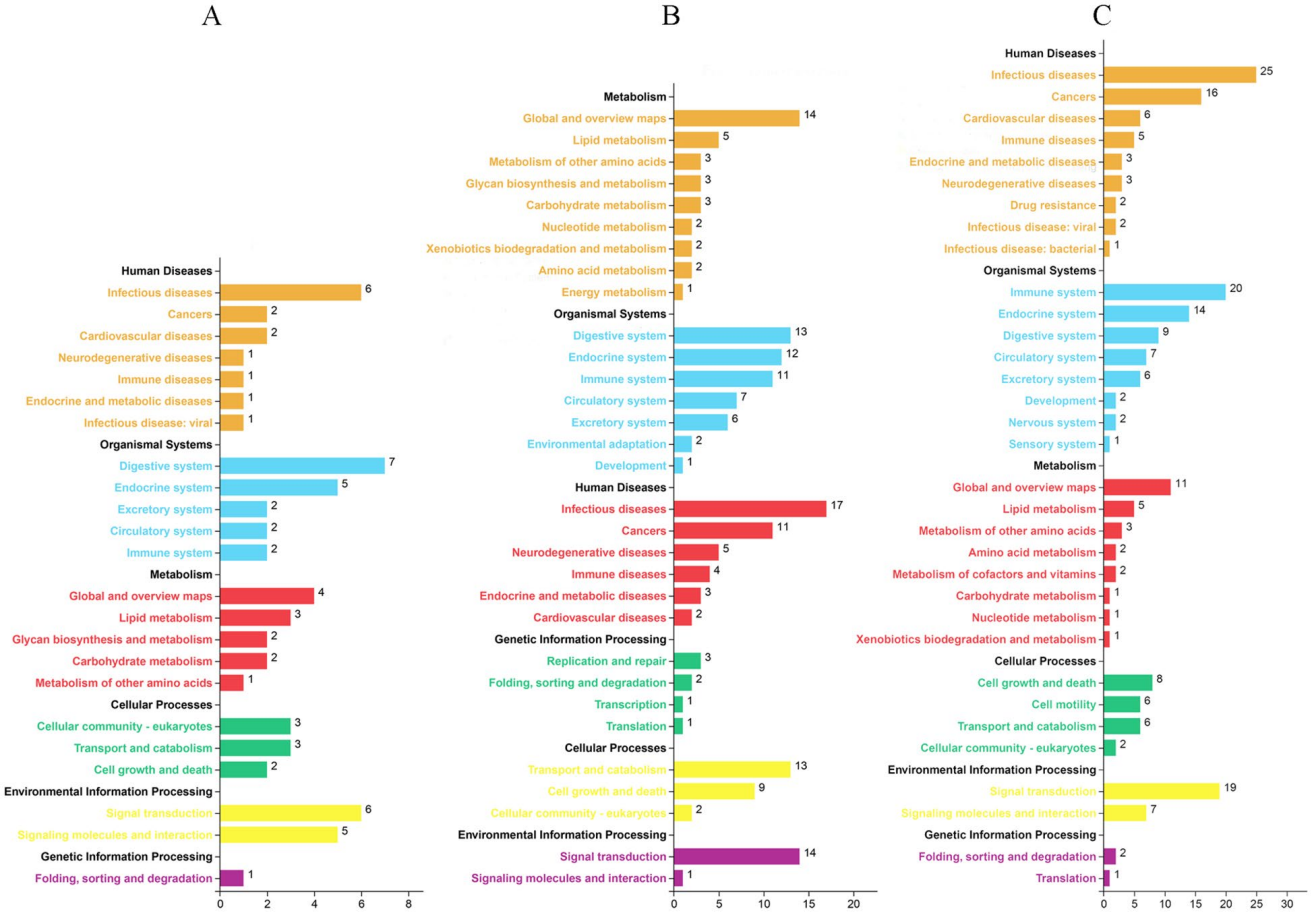
KEGG classification of differential expression genes under Alkalinity (**A**), Salinity (**B**) and Salinity–Alkalinity stresses (**C**). Numbers on column chart represent the number of genes enriched on different KEGG classes

Pairwise comparisons were performed to explore proteins associated with different stresses. The statistical significance of the observed FC was determined by paired t-tests for all DEPs, and the thresholds of *q-value* ≤ 0.05 and FC ≥ 1.5 were used. Compared with the freshwater group, we found 36, 144, and 113 DEPs in the Alkaline, Saline, and Saline–alkaline groups, respectively (Fig. 2) and DEPs enrichment differences (Fig. 3).

In the alkaline group, 36 DEPs comprised 25 up-regulated proteins and 11 down-regulated proteins. Relative to other stimuli, fewer DEPs were identified in the alkaline group. These up-regulated DEPs were dramatically enriched in the vacuole (GO:0,005,773, *p-adjust* = 0.043516) and membrane-bounded vesicle (GO:0,031,988, *p-adjust* = 0.043516). Their molecular function was significantly related to various hydrolase activities (GO:0,004,553, *p-adjust* = 0.015420; GO:0,004,559, *p-adjust* = 0.015420; GO:0,004,308, *p-adjust* = 0.028848) and ion transmembrane transporter activity (GO:0,015,079, *p-adjust* = 0.020365; GO:0,042,625, *p-adjust* = 0.037908). However, these up-regulated genes were not enriched into biological processes. For the 11 down-regulated proteins, no significantly enriched items were found (Additional file 1: Table S2).

Under saline stress, the 144 DEPs comprised 65 up-regulated DEPs and 79 down-regulated DEPs. The up-regulated DEPs were mainly dramatically enriched in the extracellular region (GO:0,005,576, *p-adjust* = 0.029859), which was related to the molecular functions of proteins in the enriched items of transmembrane transport of substances (GO:0,008,556, *p-adjust* = 0.000001; GO:0,019,829, *p-adjust* = 0.000015; GO:0,022,892, *p-adjust* = 0.000285). Clearly, these DEPs participated in homeostatic processes (GO:0,042,592, *p-adjust* = 0.010835). In addition, the DEPs were also concentrated in the regulation of the hormone metabolic process (GO:0,032,351, *p-adjust* = 0.000109) and male gamete generation process (GO:0,048,232, *p-adjust* = 0.006783), which were related to the molecular function of multiple metabolic enzyme activities (GO:0,004,866, *p-adjust* = 0.030173; GO:0,016,853, *p-adjust* = 0.033649; GO:0,016,972, *p-adjust* = 0.034029). For the 79 down-regulated DEPs, these genes were only enriched in the lytic vacuole (GO:0,000,323, *p-adjust* = 0.044831), implying a decreased activity of various lyases (Additional file 1: Table S3).

In the Saline–alkaline group, 59 up-regulated proteins and 54 down-regulated proteins were isolated. GO analysis showed that these up-regulated DEPs were especially enriched in biological processes including ion transportation (GO:0,010,107, *p-adjust* = 0.000000; GO:0,071,436, *p-adjust* = 0.000000; GO:0,098,655, *p-adjust* = 0.000380; GO:0,098,660, *p-adjust* = 0.000380), the regulation of hormone and response processes (GO:0,032,351, *p-adjust* = 0.000001; GO:0,009,725, *p-adjust* = 0.001909), organism reproduction (GO:0,032,504, *p-adjust* = 0.000159; GO:0,048,232, *p-adjust* = 0.000204; GO:0,019,953, *p-adjust* = 0.004815), muscle movement (GO:0,090,075, *p-adjust* = 0.000063; GO:0,001,508, *p-adjust* = 0.000197), the cellular response to endogenous stimulus (GO:0,071,495, *p-adjust* = 0.005024; GO:0,034,614, *p-adjust* = 0.011906; GO:0,010,033, *p-adjust* = 0.014436), fatty acid and derivative biosynthetic process and transport (GO:0,042,304, *p-adjust* = 0.020638; GO:1,901,570, *p-adjust* = 0.020638). The matching molecular functions are listed in Additional file 1: Table S4. However, we did not find significantly enriched items for the 54 down-regulated proteins.

Meanwhile, KEGG analyses were also performed to identify similarities and differences among DEPs uniquely associated with saline stress, alkaline stress, and saline–alkaline stress. Compared with alkaline and saline groups, more terms in saline–alkaline group were enriched in immune system and signal transduction (Fig. 3). The 36, 144, and 113 DEPs in the respective Alkaline, Saline and Saline–alkaline groups were annotated in the KEGG database and were grouped into 11, 19, and 24 pathway terms with *q-value* ≤ 0.05 (Additional file 1: Table S5). We found that complement and coagulation cascades (ko04610, *q-value* = 0.028015), arachidonic acid metabolism (ko00590, *q-value* = 0.022387), the cGMP-PKG signaling pathway (ko04022, *q-value* = 0.027601), thyroid hormone signaling pathway (ko04919, *q-value* = 0.030550), and adrenergic signaling in cardiomyocytes (ko04261, *q-value* = 0.032128) were uniquely annotated in the Saline–alkaline group. These GO and KEGG items enriched in alkaline and saline groups indicated that the effect of saline–alkaline stress involves more than the combination of saline and alkaline stresses.

### Gene regulated network

To explore the expression patterns and potential regulatory associations under different stimuli, we constructed a network diagram of protein interaction relationships using Cytoscape and the STRING protein interaction database. Within the network, the top hub genes of the DEPs in saline and saline–alkaline stress were isolated using the Cytohubba App in Cytoscape.

**Fig. 4:**
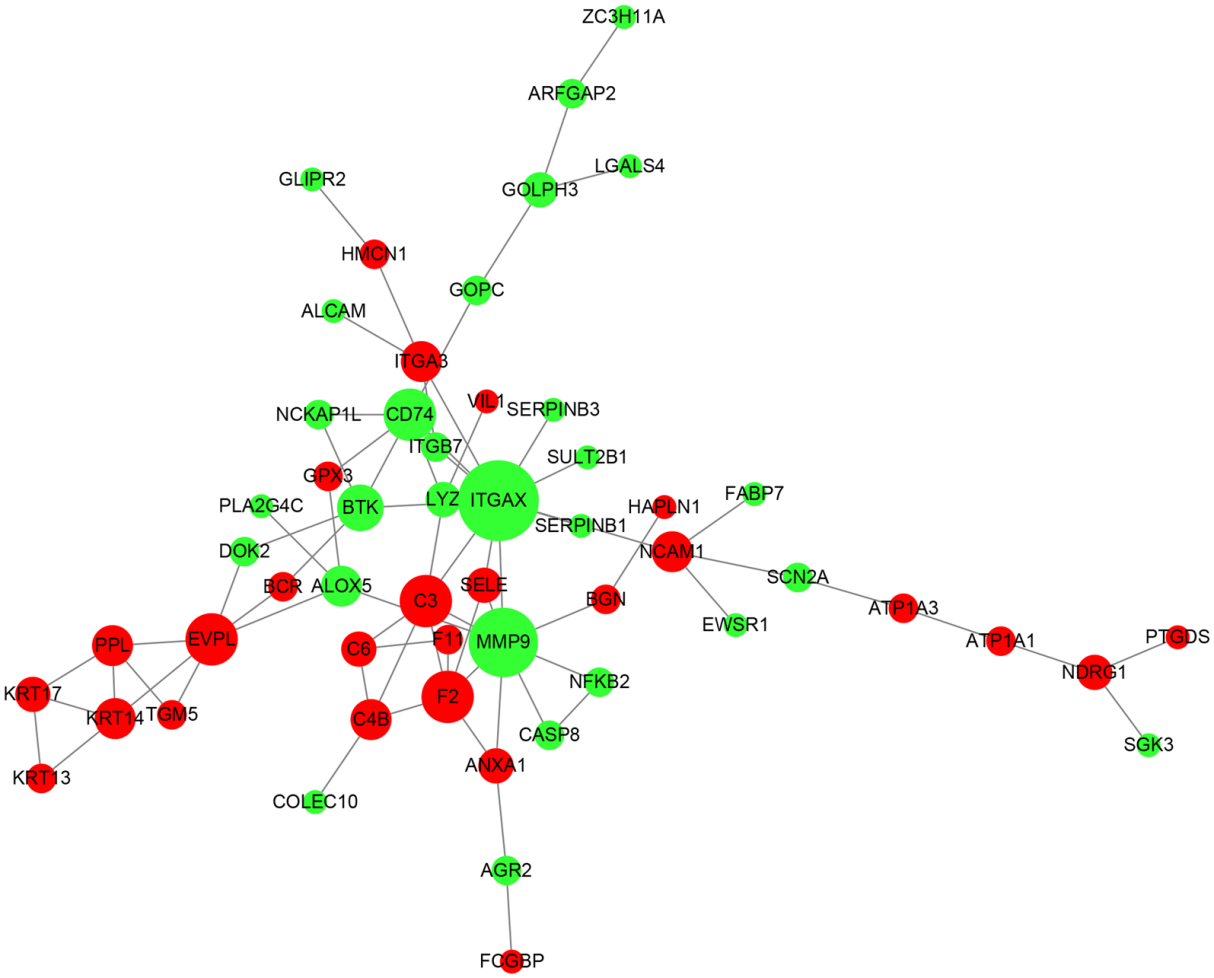
Regulated network analysis of saline–alkaline stress induced genes. Red and green nodes in the network present up-regulation or downregulation of proteins. Names of the nodes are symbols of proteins. The size of a node is proportional to its degree. Nodes with higher degrees, which means having more neighbors, will have a stronger capacity to modulate adjacent genes than genes with lower degrees

For the saline–alkaline stress network, Cytohubba isolated the genes symbolized as MMP9, ITGAX, C3, F2, CD74, EVPL, KRT17, SELE, C4B, PPL, KRT14, BTK, NCAM1, ALOX5, SERPINB1, SERPINB3, SULT2B1, ITGB7, SCN2A, KRT13, TGM5, NFKB2, CASP8 and NCKAP1L (Fig. 4). These genes were found to be significantly associated with the cell migration process (GO:0,016,477, *p-adjust* = 0.032519) and leukocyte cell–cell adhesion (GO:0,007,159, *p-adjust* = 0.032519), and played roles in MHC protein binding (GO:0,042,287, *p-adjust* = 0.035368) and signaling receptor activity (GO:0,038,023, *p-adjust* = 0.035368) in the MF category. From the KEGG analysis, the enriched KEGG pathways of immune-related activities (ko05150, *q-value* = 0.000824; ko04610, *q-value* = 0.037915) and estrogen signaling (ko04610, *q-value* = 0.023654) were related to the functions of C4B, ITGAX, Krt17, KRT14, krt13, MMP9 and F2 (Table 2).

**Table 2:** Gene Ontology (GO) and KEGG pathways classification of Salinity–Alkalinity hub genes

A. Biological process					
GO ID	Description	Salinity–alkalinity hub genes (16)	All (4696)	*p-value*	*p-adjust*
GO:0,001,667	Ameboidal-type cell migration	6 (37.5%)	160 (3.41%)	0.000009	0.006336
GO:0,051,240	Positive regulation of multicellular organismal process	5 (31.25%)	159 (3.39%)	0.000135	0.032519
GO:0,016,477	Cell migration	7 (43.75%)	392 (8.35%)	0.000158	0.032519
GO:0,007,159	Leukocyte cell–cell adhesion	4 (25%)	95 (2.02%)	0.000237	0.032519
GO:0,030,335	Positive regulation of cell migration	3 (18.75%)	41 (0.87%)	0.000320	0.032519
GO:0,051,272	Positive regulation of cellular component movement	3 (18.75%)	41 (0.87%)	0.000320	0.032519
GO:2,000,147	Positive regulation of cell motility	3 (18.75%)	41 (0.87%)	0.000320	0.032519
GO:0,048,870	Cell motility	7 (43.75%)	454 (9.67%)	0.000397	0.032519
GO:0,051,674	Localization of cell	7 (43.75%)	454 (9.67%)	0.000397	0.032519
GO:0,071,624	Positive regulation of granulocyte chemotaxis	2 (12.5%)	11 (0.23%)	0.000588	0.043341
GO:0,040,017	Positive regulation of locomotion	3 (18.75%)	53 (1.13%)	0.000685	0.045921

B. Molecular function					
GO ID	Description	Salinity–alkalinity hub genes (15)	All (4619)	*p-value*	*p-adjust*
GO:0,042,287	MHC protein binding	2 (13.33%)	14 (0.3%)	0.000876	0.035368
GO:0,038,023	Signaling receptor activity	3 (20%)	64 (1.39%)	0.001025	0.035368
GO:0,004,872	Receptor activity	3 (20%)	91 (1.97%)	0.002835	0.048320
GO:0,023,026	MHC class II protein complex binding	1 (6.67%)	1 (0.02%)	0.003247	0.048320
GO:0,004,871	Signal transducer activity	3 (20%)	98 (2.12%)	0.003501	0.048320

C. Cellular component					
GO ID	Description	Salinity–alkalinity hub genes (18)	All (4741)	*p-value*	*p-adjust*
GO:0,005,886	Plasma membrane	6 (33.33%)	462 (9.74%)	0.005549	0.225734
GO:0,071,944	Cell periphery	6 (33.33%)	488 (10.29%)	0.007269	0.225734
GO:0,005,887	Integral component of plasma membrane	2 (11.11%)	42 (0.89%)	0.010716	0.225734
GO:0,043,235	Receptor complex	2 (11.11%)	42 (0.89%)	0.010716	0.225734
GO:0,031,226	Intrinsic component of plasma membrane	2 (11.11%)	49 (1.03%)	0.014409	0.225734
GO:0,098,797	Plasma membrane protein complex	2 (11.11%)	49 (1.03%)	0.014409	0.225734
GO:0,044,421	Extracellular region part	5 (27.78%)	427 (9.01%)	0.018489	0.237034
GO:0,005,576	Extracellular region	5 (27.78%)	445 (9.39%)	0.021790	0.237034
GO:0,016,021	Integral component of membrane	4 (22.22%)	296 (6.24%)	0.022695	0.237034
GO:0,030,176	Integral component of endoplasmic reticulum membrane	1 (5.56%)	8 (0.17%)	0.029995	0.281950
GO:0,044,459	Plasma membrane part	4 (22.22%)	340 (7.17%)	0.035620	0.304391

D. KEGG pathway enrichment					
Pathway	Candidate genes with pathway annotation (19)	All genes with pathway annotation (3593)	*p-value*	*q-value*	Pathway ID
Staphylococcus aureus infection	5 (26.32%)	59 (1.64%)	0.000010	0.000824	ko05150
Estrogen signaling pathway	4 (21.05%)	76 (2.12%)	0.000563	0.023654	ko04915
Complement and coagulation cascades	3 (15.79%)	43 (1.2%)	0.001354	0.037915	ko04610
Legionellosis	3 (15.79%)	63 (1.75%)	0.004075	0.085579	ko05134

In the network constructed with DEPs under saline stress (Additional file 1: Figure S2), the hub genes identified as the top 5% of the genes using different algorithms included WDR12, EBNA1BP2, RBM19, DDX54, RPF1, SURF6, RRP1, PPL, EVPL, PKP1, CST3, C1QB, CTSS, LGMN, LGALS4, MPEG1, PTGDS, MB, CTSZ, and DRG1. From the GO analysis (in cellular components), it was observed that the hub genes were only significantly present in the lytic vacuole (*p-adjust* = 0.023081(GO:0,000,323)), being associated with the peptidase activity in the MF category and the function of extracellular matrix disassembly (GO:0,022,617, *p-adjust* = 0.032489) (Additional file 1: Table S6).

Although a network was not constructed for the alkaline stress successfully, the comparison between saline–alkaline and saline stresses indicated large differences of the response model under these factors.

### Functional categories of specific DEPs in saline–alkaline adaptation

To further understand the proteins and cellular pathways related to the saline–alkaline effect, the DEPs under different conditions were compared, and the specific DEPs related to saline–alkaline effect were isolated by Venn diagram analysis. A total of 62 specific DEPs comprising 37 down-regulated and 25 up-regulated proteins were isolated. In the enrichment analysis, the 25 up-regulated proteins were enriched in hormone synthesis and reaction items (GO:0,051,384, *p-adjust* = 0.019294), defense response (GO:0,006,952, *p-adjust* = 0.037216; GO:0,032,717, *p-adjust* = 0.019294; GO:0,031,392, *p-adjust* = 0.019294), and complement activation (GO:0,006,956, *p-adjust* = 0.045632) (Table 3). In contrast, no enriched items were found for the 37 down-regulated specifical DEPs.

**Table 3:** Gene Ontology (GO) and KEGG pathways classification of specific salinity–alkalinity up-regulated genes

A. Biological process					
GO ID	Description	Saline–alkaline up-regulated deps (18)	All (4696)	*p-value*	*p-adjust*
GO:0,048,771	Tissue remodeling	3 (16.67%)	19 (0.4%)	0.000044	0.019294
GO:0,051,384	Response to glucocorticoid	3 (16.67%)	23 (0.49%)	0.000080	0.019294
GO:0,034,614	Cellular response to reactive oxygen species	3 (16.67%)	25 (0.53%)	0.000103	0.019294
GO:0,006,816	Calcium ion transport	4 (22.22%)	85 (1.81%)	0.000252	0.019294
GO:0,010,519	Negative regulation of phospholipase activity	2 (11.11%)	8 (0.17%)	0.000383	0.019294
GO:0,032,717	Negative regulation of interleukin-8 production	2 (11.11%)	8 (0.17%)	0.000383	0.019294
GO:0,060,192	Negative regulation of lipase activity	2 (11.11%)	8 (0.17%)	0.000383	0.019294
GO:0,070,838	Divalent metal ion transport	4 (22.22%)	99 (2.11%)	0.000453	0.019294
GO:0,072,511	Divalent inorganic cation transport	4 (22.22%)	99 (2.11%)	0.000453	0.019294
GO:0,034,599	Cellular response to oxidative stress	3 (16.67%)	41 (0.87%)	0.000460	0.019294
GO:0,001,516	Prostaglandin biosynthetic process	2 (11.11%)	9 (0.19%)	0.000492	0.019294
GO:0,031,392	Regulation of prostaglandin biosynthetic process	2 (11.11%)	9 (0.19%)	0.000492	0.019294
GO:0,042,304	Regulation of fatty acid biosynthetic process	2 (11.11%)	9 (0.19%)	0.000492	0.019294
GO:0,046,456	Icosanoid biosynthetic process	2 (11.11%)	9 (0.19%)	0.000492	0.019294
GO:0,046,457	Prostanoid biosynthetic process	2 (11.11%)	9 (0.19%)	0.000492	0.019294
GO:1,901,570	Fatty acid derivative biosynthetic process	2 (11.11%)	9 (0.19%)	0.000492	0.019294
GO:2,001,279	Regulation of unsaturated fatty acid biosynthetic process	2 (11.11%)	9 (0.19%)	0.000492	0.019294
GO:1,901,701	Cellular response to oxygen-containing compound	3 (16.67%)	48 (1.02%)	0.000734	0.027216
GO:0,032,637	Interleukin-8 production	2 (11.11%)	12 (0.26%)	0.000895	0.029862
GO:0,032,677	Regulation of interleukin-8 production	2 (11.11%)	12 (0.26%)	0.000895	0.029862
GO:1,901,989	Positive regulation of cell cycle phase transition	2 (11.11%)	13 (0.28%)	0.001056	0.032011
GO:1,901,992	Positive regulation of mitotic cell cycle phase transition	2 (11.11%)	13 (0.28%)	0.001056	0.032011
GO:0,031,960	Response to corticosteroid	3 (16.67%)	57 (1.21%)	0.001216	0.034156
GO:0,032,309	Icosanoid secretion	2 (11.11%)	14 (0.3%)	0.001229	0.034156
GO:0,071,715	Icosanoid transport	2 (11.11%)	15 (0.32%)	0.001415	0.036297
GO:1,901,571	Fatty acid derivative transport	2 (11.11%)	15 (0.32%)	0.001415	0.036297
GO:0,006,979	Response to oxidative stress	4 (22.22%)	136 (2.9%)	0.001501	0.037092
GO:0,006,952	Defense response	7 (38.89%)	495 (10.54%)	0.001562	0.037216
GO:0,090,068	Positive regulation of cell cycle process	2 (11.11%)	17 (0.36%)	0.001824	0.041958
GO:0,001,776	Leukocyte homeostasis	2 (11.11%)	18 (0.38%)	0.002048	0.043514
GO:0,046,717	Acid secretion	2 (11.11%)	18 (0.38%)	0.002048	0.043514
GO:0,046,942	Carboxylic acid transport	3 (16.67%)	69 (1.47%)	0.002115	0.043514
GO:0,015,849	Organic acid transport	3 (16.67%)	70 (1.49%)	0.002205	0.043514
GO:0,006,636	Unsaturated fatty acid biosynthetic process	2 (11.11%)	19 (0.4%)	0.002283	0.043514
GO:0,045,931	Positive regulation of mitotic cell cycle	2 (11.11%)	19 (0.4%)	0.002283	0.043514
GO:0,006,693	Prostaglandin metabolic process	2 (11.11%)	20 (0.43%)	0.002531	0.045632
GO:0,006,956	Complement activation	2 (11.11%)	20 (0.43%)	0.002531	0.045632
GO:0,044,092	Negative regulation of molecular function	4 (22.22%)	159 (3.39%)	0.002671	0.046881

B. Molecular function					
GO ID	Description	Saline–alkaline up-regulated deps (18)	All (4619)	*p-value*	*p-adjust*
GO:0,004,859	Phospholipase inhibitor activity	2 (11.11%)	9 (0.19%)	0.000508	0.043946
GO:0,008,094	DNA-dependent ATPase activity	2 (11.11%)	12 (0.26%)	0.000925	0.043946
GO:0,055,102	Lipase inhibitor activity	2 (11.11%)	15 (0.32%)	0.001462	0.046287

C. Cellular component					
GO ID	Description	Saline–alkaline up-regulated deps (19)	All (4741)	*p-value*	*p-adjust*
GO:0,005,576	Extracellular region	8 (42.11%)	445 (9.39%)	0.000168	0.012632
GO:0,044,421	Extracellular region part	7 (36.84%)	427 (9.01%)	0.000884	0.033153

D. KEGG pathway enrichment					
Pathway	Candidate genes with pathway annotation (11)	All genes with pathway annotation (3593)	*P-value*	*Q -value*	Pathway ID
Neuroactive ligand–receptor interaction	2 (18.18%)	13 (0.36%)	0.000653	0.050910	ko04080
Complement and coagulation cascades	2 (18.18%)	43 (1.2%)	0.007186	0.247888	ko04610
Herpes simplex infection	3 (27.27%)	152 (4.23%)	0.009534	0.247888	ko05168
Arginine biosynthesis	1 (9.09%)	16 (0.45%)	0.047973	0.307125	ko00220

**Fig. 5:**
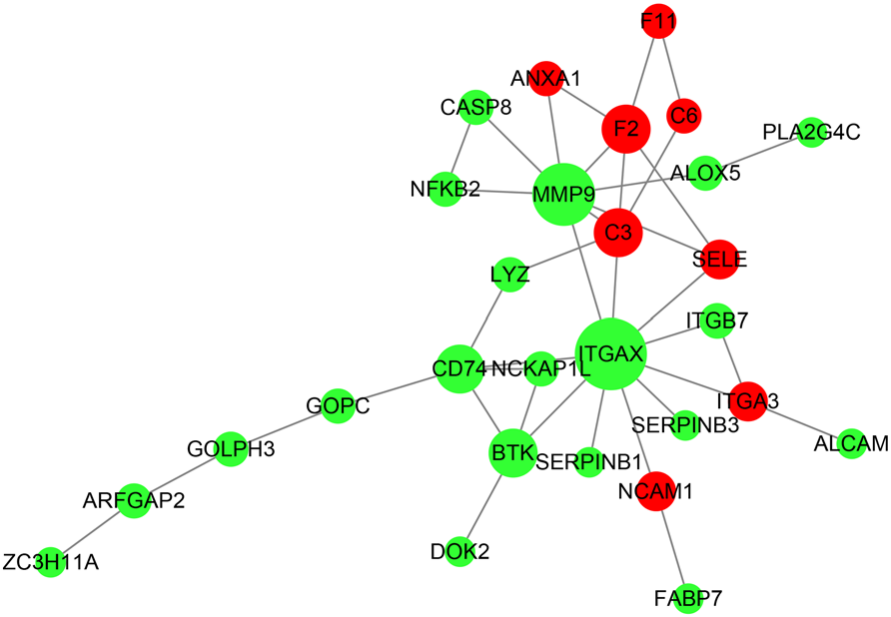
Regulated network analysis of specific saline–alkaline stress induced genes. Red and green nodes in the network present up-regulation or downregulation of proteins. Names of the nodes are symbols of proteins. The size of a node is proportional to its degree. Nodes with higher degrees, which means having more neighbors, will have a stronger capacity to modulate adjacent genes than genes with lower degrees

In the specific DEPs constructed network, the combined top 5% hub genes included ITGAX, MMP9, C3, F2, CD74, BTK, ANXA1, NCKAP1L and CASP8 (Fig. 5). Almost all the hub genes were also included in the hub genes isolated from the saline–alkaline stress network. The results indicated that the interaction of salinity and alkalinity caused independent responses during saline–alkaline stress, and that these hub genes seemed to be associated with these functions.

## Discussion

*Gymnocypris przewalskii* belongs to genus *Gymnocypris* in subfamily Schizothoracinae of Cyprinidae, and the habitat of this fish is characterized with high altitude, strong radiation, and low water temperature. Schizothoracinae fish show unique adaptability to the special environment of the plateau. The species found in China account for more than 80% of the world's total species, mainly distributed in the Qinghai-Tibet Plateau and its surrounding rivers and lakes (Chen et al. 2000; Cheng and Zheng 1987). Gill is an important organ for oxygen exchange, salinity sensing, acid–base balance, and nitrogenous waste excretion. When subjected to abiotic stress, such as saline and alkaline water, gill can initiate signal cascades to enhance the tolerance (Aruna et al. 2021; Chang et al. 2021; Li et al. 2021). Thus, gill was chosen as the research object during the analysis of stress response. DIA-SWATH method has already demonstrated its value and feasibility in model and non-model fishes (Li et al. 2018; Monroe et al. 2020; Quan et al. 2021). Due to its quantitative accuracy, dynamic range, high reproducibility, and a wide spectrum of detected peptides, we used this method to characterize the stress-responsive proteomes under alkaline, saline, and saline–alkaline interactive stresses (Frederick et al. 2016; Gillet et al. 2012). In this study, we isolated more than 7000 proteins from the gills of each treatment group. Pearson’s correlation coefficients employed in the PCA analysis indicated that the saline–alkaline group showed more difference from the other groups. This also indicated that the effect of saline–alkaline conditions on *G. przewalskii* is more complicated than that of single saline or single alkaline stresses.

Inconsistency between transcriptome and proteome

**Fig. 6:**
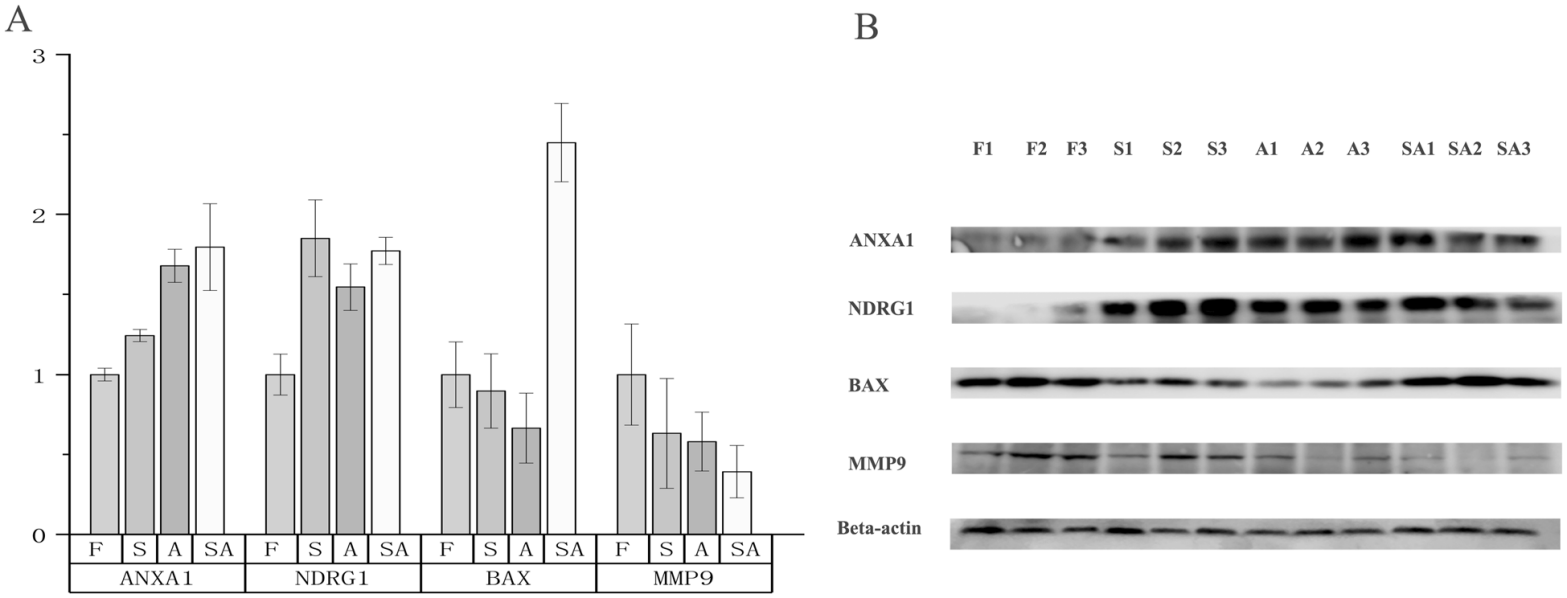
The identified differentially expressed proteins using DIA/SWATH method (**A**) and western blotting verification (**B**). S indicates replicate in Salinity stress, A for Alkalinity, F for Freshwater and SA for Salinity–Alkalinity

Transcriptomic method has been widely used for providing a global overview of gene expression patterns and pathways that are related to osmoregulation (Su et al. 2020; Xu et al. 2013a; Xu et al. 2013b; Yao et al. 2012). For *G. przewalskii,* Tong et al. (2017) found sets of genes of ion homoeostasis, acid–base balance, and innate immunity underlying their adaptation to extreme high altitude aquatic life on the Tibetan Plateau using de novo transcriptome. Su et al. (2020) provided a global overview of gene expression patterns and pathways related to osmoregulation in hybrid tilapia and elucidated the osmoregulation mechanisms related to different osmotic stresses, similar to our analysis. Although the purpose of our experiment was to explain the environmental adaptability of an indigenous fish, Su et al.'s results still provide a comparable model with which to explain the saline–alkaline adaptation process of *G. przewalskii.* However, we note that only using transcriptome data cannot illuminate the full osmotic regulation mechanism, as protein expression is a complex biological process in which changes in protein abundances of many genes are inconsistent with or even completely opposite to changes in transcript levels (Persson et al. 2009). In this study, the low Pearson's correlation coefficients of the log2 ratio of transcript and protein (0.0455 for Alkali, 0.093 for Saline–alkaline, and 0.1219 for Saline) indicated the presence of translation regulation during various stresses. In addition, Western blotting validation was performed for several genes, and the results demonstrated the reliability of the DIA-MS method (Fig. 6). At the same time, our laboratory has established muscle and gill tissue (unpublished) cell lines of *G. przewalskii*, and we believe that the functions of screened genes can be further verified.

### Saline–alkaline stress promotes protein expression in homeostasis processes

Homeostasis is related to the maintenance of an internal steady state, including physical and chemical conditions in living systems (Betts et al. 2013). Disruption of homeostasis may be the reason that juvenile naked carp’s severely hypoxia phenotype and high mortality under acute stress combining 12‰ salinity and pH 10.1 alkalinity in our lab. Under saline–alkaline stress, the DEPs enriched in metal ions homeostatic process included atp1a1 (sodium/potassium-transporting ATPase subunit alpha), atp1a3 (sodium/potassium-transporting ATPase subunit alpha) and atnb233 (sodium/potassium-transporting ATPase subunit beta-233), ANXA1 (annexin), F2 (prothrombin), rbp4a (retinol-binding Protein), Tgm5 (transglutaminase 5) and tmx2b (thioredoxin-related transmembrane protein 2B) (Table 4). Atp1a1, atp1a3, and atnb233 are subunits of Na^+^/K^+^-ATPase, a membrane protein that couples the exchange of two extracellular K^+^ ions and three intracellular Na^+^ ions accompanying hydrolysis of an ATP molecule. This pump takes part in pathways functioning as signal transducers associated with cell growth and differentiation (Li and Langhans 2015). In fish physiology, Na^+^/K^+^-ATPases in the epithelial cells of the gills and kidney play important roles in maintaining the blood ion and acid–base balance of fish in order to counter the difference in ion concentration between seawater and freshwater (Kaplan 2002; Marshall 2002). ANXA1, Tgm5, and F2 are multifunctional proteins that exhibit high affinity for calcium ions and up-regulated by the intracellular concentration of calcium (Benarous et al. 1976; Deerfield et al. 1987; Mailliard et al. 1996).

**Table 4:** Homeostasis, immune response and reproductive progress-related proteins under saline–alkaline stress in *G. przewalskii*

Gene symbol	Aligned protein description	Fold change	*q-value*	Pivotal GO process
ANXA1	Annexin A1-like isoform X1 [*Sinocyclocheilus graham*i]	1.49983	0.000733	GO:0,001,776//leukocyte homeostasis; GO:0,002,684//positive regulation of immune system process; GO:0,006,816//calcium ion transport; GO:0,030,217//T cell differentiation; GO:0,032,717//negative regulation of interleukin-8 production; GO:0,034,097//response to cytokine; GO:0,034,614//cellular response to reactive oxygen species; GO:0,043,627//response to estrogen; GO:0,051,384//response to glucocorticoid
Atp1a1	Na + /K + ATPase alpha subunit isoform 8 [*Danio rerio*]	1.789847	7.18E − 05	GO:0,006,875//cellular metal ion homeostasis; GO:0,010,107//potassium ion import; GO:0,031,944//negative regulation of glucocorticoid metabolic process; GO:0,048,232//male gamete generation; GO:0,071,436//sodium ion export; GO:1,902,600//hydrogen ion transmembrane transport
Tmx2b	Thioredoxin-related transmembrane protein 2-B-like [*Sinocyclocheilus anshuiensis*]	1.627557	0.002322	GO:0,019,725//cellular homeostasis
Rbp4a	Retinol-binding protein [Clarias batrachus]	1.632367	0.000621	GO:0,002,639//positive regulation of immunoglobulin production; GO:0,008,406//gonad development; GO:0,030,540//female genitalia development; GO:0,048,232//male gamete generation; GO:0,048,545//response to steroid hormone; GO:0,048,598//embryonic morphogenesis
Hmox	Heme oxygenase-like [Sinocyclocheilus rhinocerous]	0.578708	0.001054	GO:0,000,302//response to reactive oxygen species; GO:0,001,666//response to hypoxia; GO:0,002,262//myeloid cell homeostasis; GO:0,002,719//negative regulation of cytokine production involved in immune response; GO:0,002,887//negative regulation of myeloid leukocyte mediated immunity; GO:0,043,122//regulation of I-kappaB kinase/NF-kappaB signaling; GO:0,046,916//cellular transition metal ion homeostasis; GO:0,055,114//oxidation–reduction process
Atp1a3	Sodium/potassium-transporting ATPase subunit alpha-3 isoform X2 [Sinocyclocheilus grahami]	1.408045	0.00021	GO:0,006,875//cellular metal ion homeostasis; GO:0,010,107//potassium ion import; GO:0,015,988//energy coupled proton transmembrane transport, against electrochemical gradient; GO:0,031,944//negative regulation of glucocorticoid metabolic process; GO:0,071,436//sodium ion export
Atnb233	Sodium/potassium-transporting ATPase subunit beta-233-like [Sinocyclocheilus grahami]	1.728458	6.95E − 5	GO:0,002,376//immune system process; GO:0,006,875//cellular metal ion homeostasis; GO:0,007,599//hemostasis; GO:0,010,107//potassium ion import; GO:0,032,596//protein transport into membrane raft; GO:0,071,436//sodium ion export; GO:1,901,021//positive regulation of calcium ion transmembrane transporter activity; GO:1,901,381//positive regulation of potassium ion transmembrane transport; GO:1,902,307//positive regulation of sodium ion transmembrane transport
Gpx3	Glutathione peroxidase 3-like isoform X1 [Sinocyclocheilus grahami]	1.64715	0.00031	GO:0,006,979//response to oxidative stress; GO:0,010,033//response to organic substance; GO:0,042,743//hydrogen peroxide metabolic process; GO:0,044,710//single-organism metabolic process; GO:0,051,260//protein homooligomerization
Tgm5	Protein-glutamine gamma-glutamyltransferase 5-like [Carassius auratus]	2.229044	0.000925	GO:0,032,469//endoplasmic reticulum calcium ion homeostasis; GO:0,051,560//mitochondrial calcium ion homeostasis
Pycr2	Pyrroline-5-carboxylate reductase 1, mitochondrial-like [Sinocyclocheilus grahami]	0.576474	0.001034	GO:0,006,561//proline biosynthetic process; GO:0,006,979//response to oxidative stress; GO:0,042,391//regulation of membrane potential; GO:0,060,548//negative regulation of cell death
F2	Complement system-related protein F2-1 [Cyprinus carpio]	1.30973	0.000262	GO:0,002,376//immune system process; GO:0,010,524//positive regulation of calcium ion transport into cytosol
GPX4	Glutathione peroxidase 4a isoform 2 precursor [Danio rerio]	2.245004	0.000831	GO:0,042,743//hydrogen peroxide metabolic process; GO:0,043,627//response to estrogen; GO:0,048,232//male gamete generation
Arg2-a	Arginase-2, mitochondrial [Sinocyclocheilus rhinocerous]	2.220417	0.00258	GO:0,010,038//response to metal ion; GO:0,032,769//negative regulation of monooxygenase activity; GO:0,034,614//cellular response to reactive oxygen species; GO:0,044,702//single organism reproductive process; GO:0,044,703//multi-organism reproductive process; GO:0,071,385//cellular response to glucocorticoid stimulus
Cml	Probable N-acetyltransferase camello isoform X1 [Sinocyclocheilus grahami]	1.392688	0.000481	GO:0,050,789//regulation of biological process; GO:0,071,704//organic substance metabolic process
LOC107985021	Flocculation protein FLO11-like [Sinocyclocheilus rhinocerous]	1.724763	0.001647	GO:0,045,089//positive regulation of innate immune response; GO:0,051,607//defense response to virus
NCKAP1L	Nck-associated protein 1-like [Sinocyclocheilus anshuiensis]	0.571608	0.000981	GO:0,002,260//lymphocyte homeostasis; GO:0,030,888//regulation of B cell proliferation; GO:0,032,846//positive regulation of homeostatic process; GO:0,033,627//cell adhesion mediated by integrin; GO:0,042,129//regulation of T cell proliferation; GO:0,043,370//regulation of CD4-positive, alpha–beta T cell differentiation; GO:0,043,376//regulation of CD8-positive, alpha–beta T cell differentiation; GO:0,045,577//regulation of B cell differentiation; GO:0,045,582//positive regulation of T cell differentiation
Cd74	H-2 class II histocompatibility antigen gamma chain-like [Carassius auratus]	0.499342	0.001898	GO:0,002,478//antigen processing and presentation of exogenous peptide antigen; GO:0,010,935//regulation of macrophage cytokine production; GO:0,019,724//B cell mediated immunity; GO:0,045,058//T cell selection; GO:0,071,624//positive regulation of granulocyte chemotaxis
C6	Complement component C6 [Sinocyclocheilus grahami]	1.823065	0.001384	GO:0,006,956//complement activation
C4B	Complement system-related protein C4b [Cyprinus carpio]	1.522712	0.00071	GO:0,019,724//B cell mediated immunity; GO:0,048,583//regulation of response to stimulus
Mxb	Interferon-induced GTP-binding -like protein [Labeo rohita]	0.184448	0.00114	GO:0,002,252//immune effector process
C3	Complement C3-like [Sinocyclocheilus rhinocerous]	2.12463	0.002882	GO:0,006,956//complement activation
MMP9	Mmp-9 [Schizothorax prenanti]	0.392545	0.003211	GO:0,002,684//positive regulation of immune system process; GO:0,043,627//response to estrogen; GO:0,044,703//multi-organism reproductive process; GO:0,070,555//response to interleukin-1
Atp1a1	Na + /K + ATPase alpha subunit isoform 8 [Danio rerio]	2.719346	7.18E − 05	GO:0,048,232//male gamete generation; GO:0,048,598//embryonic morphogenesis
Rbp4a	Retinol-binding protein [Clarias batrachus]	2.276576	0.000621	GO:0,008,406//gonad development; GO:0,030,540//female genitalia development; GO:0,048,232//male gamete generation
GRN	Granulins-like isoform X3 [Sinocyclocheilus grahami]	0.658712	0.000245	GO:0,044,703//multi-organism reproductive process; GO:0,044,706//multi-multicellular organism process; GO:0,048,522//positive regulation of cellular process
MST1	Hepatocyte growth factor-like protein isoform X1 [Sinocyclocheilus rhinocerous]	1.886807	0.003166	GO:0,048,232//male gamete generation; GO:0,048,870//cell motility

Acid–base homeostasis involves the regulation of the pH of the body's extracellular fluid (ECF). A constant pH level of intracellular and extracellular fluid is crucial for the normal physiology of the body and cellular metabolism (Hamm, et al. 2015). In living things, renal ammonia metabolism and transport play central roles in acid–base homeostasis and are regulated by a variety of factors, including extracellular pH and K^+^, and by hormones such as mineralocorticoids, glucocorticoids, and angiotensin II (Sicuro et al. 1998; Weiner and Verlander 2013). Wang et al. (2003) described unusually high levels of tissue ammonia in *G. przewalskii* when cultured in lake water and suggested that high glutamate dehydrogenase and glutamine synthetase activities in tissues probably allowed the fish to alleviate ammonia toxicity via amino acid accumulation. In this study, in addition to glucocorticoid metabolic process and up-regulation of atp1a1, atp1a3, atnb233, the up-regulation of Tgm5 might also be associated with ammonia metabolism. NH_4_^+^ and HCO_3_^−^ can be used to produce urea in the liver for acid–base homeostasis (Weiner and Verlander 2016). Upregulation of Tgm5 probably takes effect by promoting NH4^+^ transportation as glutamine after NH_3_ incorporation to achieve acid–base homeostasis (Kumai, et al. 2015; Wang, et al. 2003; Yao, et al. 2016).

Redox state maintenance is an important aspect of physiological equilibrium. Alterations of this balance may cause oxidative stress that damages cells and tissues through disruption of macromolecules (Rahman 2007). Therefore, aerobic organisms are equipped with a variety of enzymatic and non-enzymatic antioxidants that are important in mediating redox balance and defending against reactive oxygen species. In this study, we observed the enrichment of DEPs related to redox functions such as GPX3 (glutathione peroxidase), GPX4 (phospholipid-hydroperoxide glutathione peroxidase), NAT8 (N-acetyltransferase 8), rbp4a, and Tmx2b (Table 4). GPX3 and GPX4 belong to the glutathione peroxidase family that catalyze the reduction of organic hydroperoxides and hydrogen peroxide (H_2_O_2_) by glutathione and thereby protect cells against oxidative damage (Margis et al. 2008). By generating glutathione (GSH), these glutathione peroxidases can act as scavengers of reactive oxygen species and regulate the oxidative state of proteins together with a transmembrane thioredoxin (Tmx2b) (Yoshihara, et al. 2010). For the NAT8 and rbp4a, these two proteins are primarily indirectly involved in the process of redox state change (Hammerling 2016; Tsuboi et al. 2002; Zanotto-Filho et al. 2008).

The isolated related proteins fully demonstrated that the maintenance of cellular homeostasis is essential to the process of adaptation to stress and the response mechanisms of cells differ according to the maintenance of homeostasis. Thus, the analysis of related cell signaling pathways can be used to understand the individual adaptation process.

### Immune response initiated by saline–alkaline stress

Immune system is very sensitive to the external environment. In general, exogenous infection, food composition, the external environment, metal elements, and even species density can activate the immune response in fish species (Bu et al. 2019; Guo et al. 2021; Huber et al. 2018; Liu et al. 2018; Rahimnejad et al. 2018; Yan et al. 2018; Zanuzzo et al. 2020). During the spawning process of anadromous fish, migration suppresses the immune system through endocrine signaling factors including growth hormone, thyroid hormone, and cortisol (Zwollo 2018). In *G. przewalskii,* Yao et al. (2012) also found that alkaline stress slowed the pathways related to the immune system (Yao et al. 2012). Although the direct cause of immunosuppression may be gonadal maturation, we believe that gonadal maturation is also associated with changes of salinity and alkalinity in anadromous fish (Carruth et al. 2000; Sandblom et al. 2009).

Under saline–alkaline stress, significantly enriched items of immune effector process (GO:0,002,252, *p-value* = 0.026632) (Additional file 1: Table S4A) included DEPs of Cd74 (CD74 Antigen), c3 (complement component 3), C6 (complement component 6), rbp4a, hmox (Heme Oxygenase), C4B (Complement Component 4B), mxb (Interferon-Regulated Resistance GTP-Binding Protein) and MMP9 (Matrix Metalloproteinase 9). From the KEGG analysis, we found the DEPs including F2 (coagulation factor II), C4 (complement component 4), C3 and ITGAM (CD11b, integrin alpha M) were significantly enriched in complement and coagulation cascades pathway (ko04610, *q-value* = 0.028015) (Additional file 1: Table S5A). Among these genes, MMP9, C3, F2, CD74, and C6 were only expressed in saline–alkaline stress. This indicated that the mixture of salinity and alkalinity dramatically promoted the complement reaction.

The complement system is responsible for various immune effecter functions, including elimination of invading pathogens, promotion of inflammatory responses, and clearance of apoptotic cells and necrotic cell debris in addition to modulation of adaptive immune responses (Nakao et al. 2011). Previous studies confirmed the activation of the thyroid pathway appeared to be stimulatory for the humoral immunity in anadromous fish (Bitencourt et al. 2012; Farkas et al. 2008; Karkhaneh et al. 2019; Liu and Lv 2008; Yilmazer et al. 2003; Zwollo 2018) and complement could be up-regulated by thyroid hormones in human hepatocellular carcinoma cell lines (Lin et al. 2003). Although the mechanism of the up-regulation of complement protein under saline–alkaline condition is unclear, we speculated that it might be related to thyroid hormone, considering the enriched pathway of thyroid hormone synthesis from the KEGG analysis (ko04918, *q-value* = 0.0000) (Additional file 1: Table S5A). In general, the increase of complement levels is an important feature of the altered immune response of *G. przewalskii* under saline–alkaline stress, also a response that may have evolved during the long-term saline–alkaline adaptation process (Tong, et al. 2017).

### Relationship between reproductive development and saline–alkaline stress

Under artificial cultivation, we found that the gonad of *G. przewalskii* could not fully develop. Thus, we have considered whether artificial reproduction could be achieved with human intervention. The enriched items with up-regulated DEPs under saline–alkaline stress in reproductive development and pathways related to estrogen signaling prompted us to consider the relationship between reproductive development and saline–alkaline stress. There was no evidence to indicate that the direct relationship between stress and reproduction. However, some studies have shown changes in reproductive development under stress (Powell et al. 2021). The reproductive process is regulated by hormones, among which cortisol levels (a type of glucocorticoid) seem to play a key role, as they are increased dramatically during the spawning journey (Mccormick et al. 2013; Saha et al. 2002). The enriched item of negative regulation of glucocorticoid metabolic process (GO:0,031,944, *p-value* = 0.000001) (Additional file 1: Table S4A) using DEPs of saline–alkaline stress also implied the involvement of cortisol in the reproductive process.

Cortisol levels are related to the production of eicosanoids that are stimulated by cAMP signaling (Martins et al. 2013). In the GO and KEGG analyses, we also found the enriched items of icosanoid biosynthetic process (GO:0,046,456, *p-value* = 0.020638), arachidonic acid metabolism (ko00590, *q-value* = 0.022387) and cAMP signaling pathway (ko04024, *q-value* = 0.022387) (Table 3). Thus, changes in reproductive development under saline–alkaline stress may be result of hormonal fluctuations related to in vitro environmental stresses. Table 4 shows the GO items and pathways related to reproduction using DEPs under saline–alkaline stress. The DEPs included Itga3 (integrin subunit alpha 3), ANXA1, atp1a1, rbp4a, MST1, GPX4, arg2-a (arginase 2), KRT13 (keratin 13), KRT14 (keratin 14), KRT17 (keratin 17). Nearly half of the DEPs also functioned in homeostasis processes or the immune system. We speculated that is the reason why reproductive development was also affected by the stresses.

## Conclusions

In this study, the DIA-MS method was used to examine the physiology of saline–alkaline adaptation of *G. przewalskii* through exploring the main functional genes and the signal regulatory pathways involved in the functional genes. This method provided more accurate objects of study in compared with the transcriptome method. We found the maintenance of cellular homeostasis is essential to the process of adaptation to stress, meanwhile, DEPs were enriched significantly in Ions homeostatic process, Acid–base homeostasis and Redox state maintenance. In addition, complement system was promoted dramatically as well, that might relate with altered hormone levels induced by salinity and alkalinity stresses. Similarly, probably due to the changed hormone levels, reproductive promotion might be another important consequence during saline–alkali adaptation. This research has improved our understanding of the molecular mechanisms of osmoregulation of the naked carp in response to saline–alkaline challenge and has provided insights into the adaptive responses to the combined impact of saline and alkaline conditions.

### Supplementary Information

**Additional file 1: Figure S1.** Transcriptomic identification and sample relationship analysis. **Figure S2.** Regulated network analysis of saline stress induced genes. **Table S1.** Mortality after combined stresses of salinity and alkalinit. **Table S2.** GO Enrichment of Alkalinity upregulated genes. **Table S3.** GO Enrichment of Salinity upregulated and downregulated genes. **Table S4.** GO Enrichment of Salinity-Alkalinity upregulated genes. **Table S5.** Pathway Enrichment of differentially expressed proteins under stresses. **Table S6.** GO and pathway enrichment of hub genes under salinity stress.

**Additional file 2: **Background genelist for GO annotation.

**Additional file 3: **Background genelist for KEGG analyses.

## Data Availability

The data sets supporting the results of this article are included within the article and its additional files. The reported sequences data were archived in the Sequence Read Archive (SRA) with accession number PRJNA833655. The mass spectrometry proteomics data have been deposited to iProX with the dataset identifier PXD033567. Background genelists for GO and KEGG enrichment analysis were supplied as additional files.
