# Supplementary material for: Transcriptomic and proteomic analyses provide insights into the adaptive responses to the combined impact of salinity and alkalinity in Gymnocypris przewalskii
Source: Bioresour Bioprocess. 2022 Sep 26;9(1):104. doi: 10.1186/s40643-022-00589-1 (PMC10992934; doi:10.1186/s40643-022-00589-1)
Supplement: Supplementary file 1 — Additional file 1: Figure S1. Transcriptomic identification and sample relationship analysis. Figure S2. Regulated network analysis of saline stress induced genes. Table S1. Mortality after combined stresses of salinity and alkalinit. Table S2. GO Enrichment of Alkalinity upregulated genes. Table S3. GO Enrichment of Salinity upregulated and downregulated genes. Table S4. GO Enrichment of Salinity-Alkalinity upregulated genes. Table S5. Pathway Enrichment of differentially expressed proteins under stresses. Table S6. GO and pathway enrichment of hub genes under salinity stress. [file 40643_2022_589_MOESM1_ESM.pdf]

A

Genes Num	79152
GC percentage	43.3128
N50 number	9979
N50 length	2328
Max length	27026
Min length	201
Average length	1041
Annotation genes	37886
Without annotation genes	41266

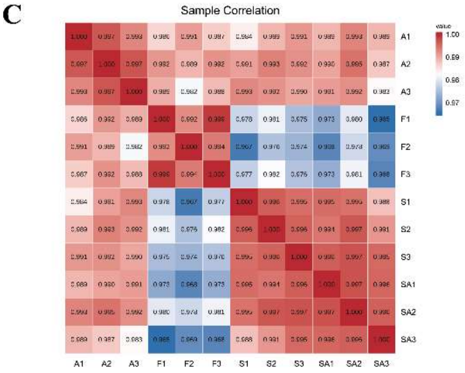

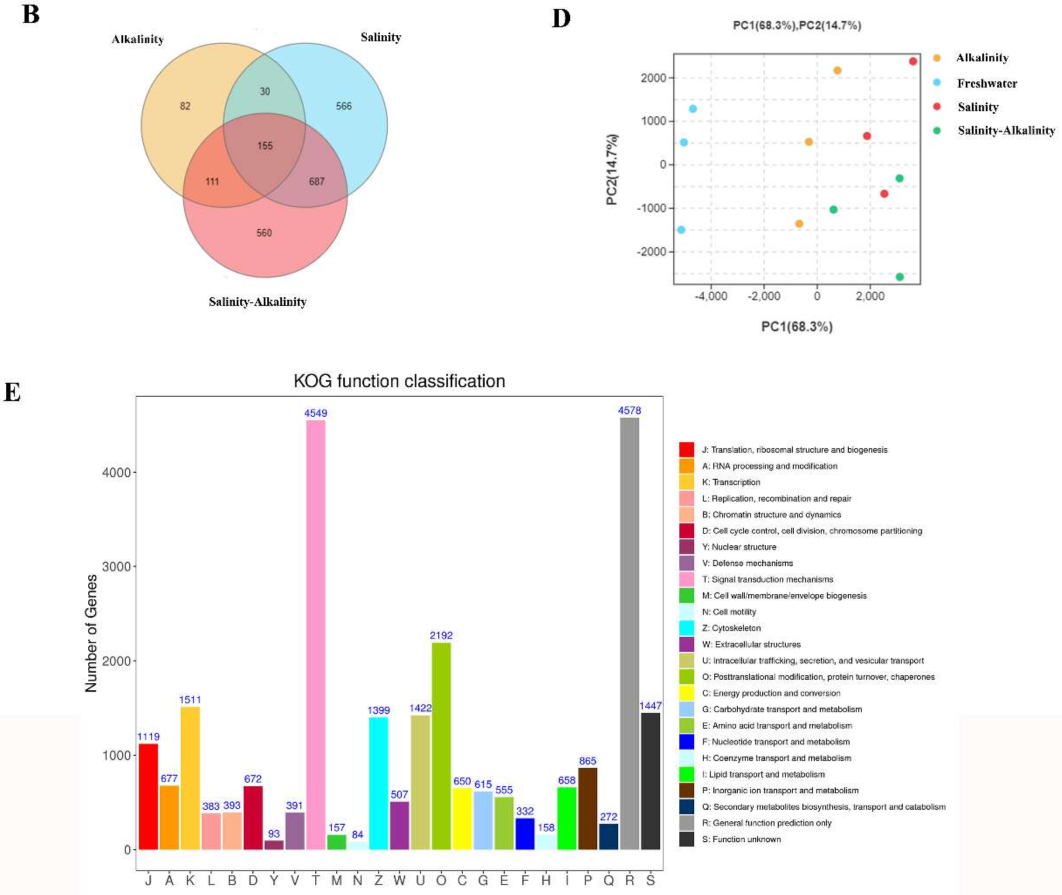

Figure S1. Transcriptomic identification and sample relationship analysis. (A) Assembly quality and annotation statistics of transcriptome. (B) Venn diagram of overlap of the differentially expressed genes under stresses. (C) Correlation analysis among different groups; (D) PCA of different individuals. F indicated freshwater group, S for salinity, A for alkalinity, SA for salinity-alkalinity. (E) KOG functional classification of the total genes.

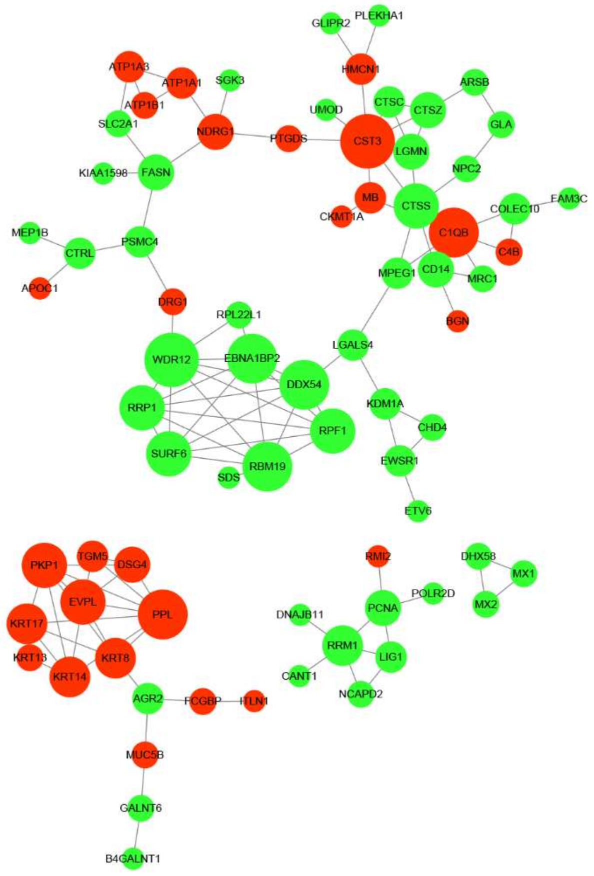

Figure S2. Regulated network analysis of saline stress induced genes. Green and red nodes in the network present downregulated and upregulated transcription. The size of a node is proportional to its degree. Nodes with higher degrees, which means having more neighbours, will have a stronger capacity to modulate adjacent genes than genes with lower degrees.

Table S1. Mortality after combined stresses of salinity and alkalinity

Group	Salinity and alkalinity	Formulation	Mortality after stress (Means ± SE)
1	Fresh water		0%
2	8‰ salinity	8‰ sodium chloride	0 %
3	12‰ salinity	12‰ sodium chloride	0%
4	16‰ salinity	16‰ sodium chloride	1.1 ± 1.1 %
5	pH 9.2 alkalinity	0.01M carbonate buffer (pH 9.2)	0 %
6	pH 10.1 alkalinity	0.01M carbonate buffer (pH 10.1)	1.1 ± 1.1 %
7	pH 10.7 alkalinity	0.01M carbonate buffer (pH 10.7)	2.2 ± 2.2 %
8	12‰ salinity, pH 10.7 alkalinity	0.01M carbonate buffer (pH 10.7) plus 11‰ sodium chloride	44 ± 6.8 %
9	12‰ salinity, pH 10.1 alkalinity	0.01M carbonate buffer (pH 10.1) plus 11‰ sodium chloride	38 ± 8.0 %
10	12‰ salinity, pH 9.2 alkalinity	0.01M carbonate buffer (pH 9.2) plus 11‰ sodium chloride	57 ± 12.0 %

Table S2. GO Enrichment of Alkalinity upregulated genes

A. Biological Process

#	GO ID	Description	Freshwater vs Alkalinity up (13)	All (4696)	*p-value*	*p-adjust*
1	GO:0051291	protein heterooligomerization	2 (15.38%)	10 (0.21%)	0.000314	0.054971
2	GO:0010107	potassium ion import	2 (15.38%)	12 (0.26%)	0.000460	0.054971
3	GO:0086011	membrane repolarization during action potential	2 (15.38%)	14 (0.3%)	0.000632	0.054971
4	GO:0071436	sodium ion export	2 (15.38%)	15 (0.32%)	0.000728	0.054971
5	GO:0071804	cellular potassium ion transport	2 (15.38%)	19 (0.4%)	0.001178	0.054971
6	GO:0071805	potassium ion transmembrane transport	2 (15.38%)	19 (0.4%)	0.001178	0.054971
7	GO:0086009	membrane repolarization	2 (15.38%)	19 (0.4%)	0.001178	0.054971
8	GO:0006664	glycolipid metabolic process	2 (15.38%)	21 (0.45%)	0.001442	0.054971
9	GO:1903509	liposaccharide metabolic process	2 (15.38%)	21 (0.45%)	0.001442	0.054971
10	GO:0010644	cell communication by electrical coupling	2 (15.38%)	24 (0.51%)	0.001887	0.063309
11	GO:0090075	relaxation of muscle	2 (15.38%)	25 (0.53%)	0.002048	0.063309
12	GO:0035725	sodium ion transmembrane transport	2 (15.38%)	26 (0.55%)	0.002215	0.063309
13	GO:0032963	collagen metabolic process	2 (15.38%)	31 (0.66%)	0.003144	0.080673
14	GO:0001508	action potential	2 (15.38%)	33 (0.7%)	0.003559	0.080673
15	GO:0019725	cellular homeostasis	4 (30.77%)	248 (5.28%)	0.003717	0.080673
16	GO:0006813	potassium ion transport	2 (15.38%)	35 (0.75%)	0.003998	0.080673
17	GO:0044259	multicellular organismal macromolecule metabolic process	2 (15.38%)	35 (0.75%)	0.003998	0.080673
18	GO:0044236	multicellular organism metabolic process	2 (15.38%)	37 (0.79%)	0.004462	0.085017
19	GO:0032594	protein transport within lipid bilayer	1 (7.69%)	2 (0.04%)	0.005530	0.090316
20	GO:0032596	protein transport into membrane raft	1 (7.69%)	2 (0.04%)	0.005530	0.090316
21	GO:1903044	protein localization to membrane raft	1 (7.69%)	2 (0.04%)	0.005530	0.090316
22	GO:0006643	membrane lipid metabolic process	2 (15.38%)	49 (1.04%)	0.007732	0.120546
23	GO:0006875	cellular metal ion homeostasis	3 (23.08%)	161 (3.43%)	0.008781	0.130949
24	GO:0030003	cellular cation homeostasis	3 (23.08%)	167 (3.56%)	0.009712	0.138797
25	GO:0006814	sodium ion transport	2 (15.38%)	57 (1.21%)	0.010363	0.139328
26	GO:0006873	cellular ion homeostasis	3 (23.08%)	173 (3.68%)	0.010699	0.139328
27	GO:0043062	extracellular structure organization	3 (23.08%)	177 (3.77%)	0.011389	0.139328
28	GO:0006487	protein N-linked glycosylation	2 (15.38%)	60 (1.28%)	0.011439	0.139328
29	GO:0055082	cellular chemical homeostasis	3 (23.08%)	187 (3.98%)	0.013228	0.139328
30	GO:0098655	cation transmembrane transport	2 (15.38%)	66 (1.41%)	0.013734	0.139328
31	GO:0098660	inorganic ion transmembrane transport	2 (15.38%)	66 (1.41%)	0.013734	0.139328
32	GO:0098662	inorganic cation transmembrane transport	2 (15.38%)	66 (1.41%)	0.013734	0.139328
33	GO:1902307	positive regulation of sodium ion transmembrane transport	1 (7.69%)	5 (0.11%)	0.013771	0.139328
34	GO:0055065	metal ion homeostasis	3 (23.08%)	190 (4.05%)	0.013811	0.139328
35	GO:0044710	single-organism metabolic process	9 (69.23%)	1676 (35.69%)	0.014350	0.140628

36	GO:0042391	regulation of membrane potential	2 (15.38%)	71 (1.51%)	0.015787	0.150414
37	GO:0055080	cation homeostasis	3 (23.08%)	204 (4.34%)	0.016730	0.155092
38	GO:0098771	inorganic ion homeostasis	3 (23.08%)	210 (4.47%)	0.018081	0.163209
39	GO:0043268	positive regulation of potassium ion transport	1 (7.69%)	7 (0.15%)	0.019230	0.164899
40	GO:1901381	positive regulation of potassium ion transmembrane transport	1 (7.69%)	7 (0.15%)	0.019230	0.164899
41	GO:0051259	protein oligomerization	3 (23.08%)	218 (4.64%)	0.019978	0.167133
42	GO:0050801	ion homeostasis	3 (23.08%)	220 (4.68%)	0.020469	0.167165
43	GO:1901021	positive regulation of calcium ion transmembrane transporter activity	1 (7.69%)	8 (0.17%)	0.021949	0.171105
44	GO:1904427	positive regulation of calcium ion transmembrane transport	1 (7.69%)	8 (0.17%)	0.021949	0.171105
45	GO:0010765	positive regulation of sodium ion transport	1 (7.69%)	9 (0.19%)	0.024661	0.176227
46	GO:0031944	negative regulation of glucocorticoid metabolic process	1 (7.69%)	9 (0.19%)	0.024661	0.176227
47	GO:0032351	negative regulation of hormone metabolic process	1 (7.69%)	9 (0.19%)	0.024661	0.176227
48	GO:1901379	regulation of potassium ion transmembrane transport	1 (7.69%)	9 (0.19%)	0.024661	0.176227
49	GO:0006486	protein glycosylation	2 (15.38%)	95 (2.02%)	0.027324	0.176926
50	GO:0043413	macromolecule glycosylation	2 (15.38%)	95 (2.02%)	0.027324	0.176926
51	GO:0008211	glucocorticoid metabolic process	1 (7.69%)	10 (0.21%)	0.027367	0.176926
52	GO:0031943	regulation of glucocorticoid metabolic process	1 (7.69%)	10 (0.21%)	0.027367	0.176926
53	GO:0045939	negative regulation of steroid metabolic process	1 (7.69%)	10 (0.21%)	0.027367	0.176926
54	GO:0070085	glycosylation	2 (15.38%)	97 (2.07%)	0.028405	0.176926
55	GO:0032350	regulation of hormone metabolic process	1 (7.69%)	11 (0.23%)	0.030065	0.176926
56	GO:0006629	lipid metabolic process	4 (30.77%)	462 (9.84%)	0.032189	0.176926
57	GO:0042592	homeostatic process	4 (30.77%)	462 (9.84%)	0.032189	0.176926
58	GO:0045833	negative regulation of lipid metabolic process	1 (7.69%)	12 (0.26%)	0.032756	0.176926
59	GO:0060973	cell migration involved in heart development	1 (7.69%)	12 (0.26%)	0.032756	0.176926
60	GO:1902305	regulation of sodium ion transmembrane transport	1 (7.69%)	12 (0.26%)	0.032756	0.176926
61	GO:1904064	positive regulation of cation transmembrane transport	1 (7.69%)	12 (0.26%)	0.032756	0.176926
62	GO:0006941	striated muscle contraction	2 (15.38%)	105 (2.24%)	0.032897	0.176926
63	GO:0009101	glycoprotein biosynthetic process	2 (15.38%)	105 (2.24%)	0.032897	0.176926
64	GO:0044723	single-organism carbohydrate metabolic process	3 (23.08%)	264 (5.62%)	0.033012	0.176926
65	GO:0051928	positive regulation of calcium ion transport	1 (7.69%)	13 (0.28%)	0.035441	0.187019

66	GO:0009311	oligosaccharide metabolic process	1 (7.69%)	14 (0.3%)	0.038119	0.198101
67	GO:0005975	carbohydrate metabolic process	3 (23.08%)	286 (6.09%)	0.040536	0.205746
68	GO:0055117	regulation of cardiac muscle contraction	1 (7.69%)	15 (0.32%)	0.040789	0.205746
69	GO:0048878	chemical homeostasis	3 (23.08%)	292 (6.22%)	0.042732	0.212424
70	GO:0009100	glycoprotein metabolic process	2 (15.38%)	127 (2.7%)	0.046586	0.228269
71	GO:0010828	positive regulation of glucose transport	1 (7.69%)	18 (0.38%)	0.048761	0.235561

B. Molecular Function

	GO ID	Description	Freshwater vs Alkalinity UP (13)	All (4619)	*p-value*	*p-adjust*
1	GO:0004553	hydrolase activity, hydrolyzing O-glycosyl compounds	3 (23.08%)	48 (1.04%)	0.000280	0.015420
2	GO:0016798	hydrolase activity, acting on glycosyl bonds	3 (23.08%)	63 (1.36%)	0.000628	0.015420
3	GO:0004559	alpha-mannosidase activity	2 (15.38%)	15 (0.32%)	0.000752	0.015420
4	GO:0015923	mannosidase activity	2 (15.38%)	15 (0.32%)	0.000752	0.015420
5	GO:0008556	potassium-transporting ATPase activity	2 (15.38%)	20 (0.43%)	0.001350	0.020365
6	GO:0015079	potassium ion transmembrane transporter activity	2 (15.38%)	21 (0.45%)	0.001490	0.020365
7	GO:0004308	exo-alpha-sialidase activity	1 (7.69%)	1 (0.02%)	0.002814	0.028848
8	GO:0016997	alpha-sialidase activity	1 (7.69%)	1 (0.02%)	0.002814	0.028848
9	GO:0015662	ATPase activity, coupled to transmembrane movement of ions, phosphorylative mechanism	2 (15.38%)	32 (0.69%)	0.003459	0.031512
10	GO:0019829	cation-transporting ATPase activity	2 (15.38%)	37 (0.8%)	0.004607	0.037780
11	GO:0042625	ATPase coupled ion transmembrane transporter activity	2 (15.38%)	41 (0.89%)	0.005637	0.037908
12	GO:0016820	hydrolase activity, acting on acid anhydrides, catalyzing transmembrane movement of substances	2 (15.38%)	44 (0.95%)	0.006472	0.037908
13	GO:0042626	ATPase activity, coupled to transmembrane movement of substances	2 (15.38%)	44 (0.95%)	0.006472	0.037908
14	GO:0043492	ATPase activity, coupled to movement of substances	2 (15.38%)	44 (0.95%)	0.006472	0.037908
15	GO:0046873	metal ion transmembrane	2 (15.38%)	47 (1.02%)	0.00736	0.040042

		transporter activity			1	
16	GO:0015399	primary active transmembrane transporter activity	2 (15.38%)	50 (1.08%)	0.008301	0.040042
17	GO:0015405	P-P-bond-hydrolysis-driven transmembrane transporter activity	2 (15.38%)	50 (1.08%)	0.008301	0.040042

C. Cellular Component

#	GO ID	Description	Freshwater vs Alkalinity UP (13)	All (4741)	*p-value*	*p-adjust*
1	GO:0005773	vacuole	4 (30.77%)	195 (4.11%)	0.0015	**0.0435**
2	GO:0031988	membrane-bounded vesicle	10 (76.92%)	1701 (35.88%)	0.0031	**0.0435**
3	GO:0000323	lytic vacuole	3 (23.08%)	114 (2.4%)	0.0032	**0.0435**
4	GO:0031982	vesicle	10 (76.92%)	1739 (36.68%)	0.0037	**0.0435**
5	GO:0044437	vacuolar part	3 (23.08%)	123 (2.59%)	0.004	**0.0435**
6	GO:0005775	vacuolar lumen	2 (15.38%)	42 (0.89%)	0.0056	0.0506
7	GO:0005886	plasma membrane	4 (30.77%)	462 (9.74%)	0.0312	0.2039
8	GO:0071944	cell periphery	4 (30.77%)	488 (10.29%)	0.0373	0.2039
9	GO:0005903	brush border	1 (7.69%)	14 (0.3%)	0.0378	0.2039
10	GO:0098862	cluster of actin-based cell projections	1 (7.69%)	14 (0.3%)	0.0378	0.2039

Table S3. GO Enrichment of Salinity upregulated and downregulated genes

A. Biological Process of upregulated genes

**#**	**GO ID**	**Description**	**Freshwater vs Salinity UP (47)**	**All (4696)**	***p-value***	***p-adjust***
1	GO:0071436	sodium ion export	6 (12.77%)	15 (0.32%)	0.000000	**0.000003**
2	GO:0071804	cellular potassium ion transport	6 (12.77%)	19 (0.4%)	0.000000	**0.000005**
3	GO:0071805	potassium ion transmembrane transport	6 (12.77%)	19 (0.4%)	0.000000	**0.000005**
4	GO:0010107	potassium ion import	5 (10.64%)	12 (0.26%)	0.000000	**0.000012**
5	GO:0035725	sodium ion transmembrane transport	6 (12.77%)	26 (0.55%)	0.000000	**0.000023**
6	GO:0006813	potassium ion transport	6 (12.77%)	35 (0.75%)	0.000001	**0.000109**
7	GO:0031944	negative regulation of glucocorticoid metabolic process	4 (8.51%)	9 (0.19%)	0.000001	**0.000109**
8	GO:0032351	negative regulation of hormone metabolic process	4 (8.51%)	9 (0.19%)	0.000001	**0.000109**
9	GO:0008211	glucocorticoid metabolic process	4 (8.51%)	10 (0.21%)	0.000002	**0.000131**
10	GO:0031943	regulation of glucocorticoid metabolic process	4 (8.51%)	10 (0.21%)	0.000002	**0.000131**
11	GO:0045939	negative regulation of steroid metabolic process	4 (8.51%)	10 (0.21%)	0.000002	**0.000131**
12	GO:0032350	regulation of hormone metabolic process	4 (8.51%)	11 (0.23%)	0.000003	**0.000186**
13	GO:0010644	cell communication by electrical coupling	5 (10.64%)	24 (0.51%)	0.000003	**0.000186**
14	GO:0090075	relaxation of muscle	5 (10.64%)	25 (0.53%)	0.000004	**0.000215**
15	GO:0045833	negative regulation of lipid metabolic process	4 (8.51%)	12 (0.26%)	0.000004	**0.000223**
16	GO:0086011	membrane repolarization during action potential	4 (8.51%)	14 (0.3%)	0.000008	**0.000417**
17	GO:0006814	sodium ion transport	6 (12.77%)	57 (1.21%)	0.000018	**0.000856**
18	GO:0086009	membrane repolarization	4 (8.51%)	19 (0.4%)	0.000031	**0.001384**
19	GO:0098655	cation transmembrane transport	6 (12.77%)	66 (1.41%)	0.000042	**0.001621**
20	GO:0098660	inorganic ion transmembrane transport	6 (12.77%)	66 (1.41%)	0.000042	**0.001621**
21	GO:0098662	inorganic cation transmembrane transport	6 (12.77%)	66 (1.41%)	0.000042	**0.001621**
22	GO:0015672	monovalent inorganic cation transport	8 (17.02%)	137 (2.92%)	0.000051	**0.001904**

23	GO:0019218	regulation of steroid metabolic process	4 (8.51%)	23 (0.49%)	0.000068	**0.002337**
24	GO:0006941	striated muscle contraction	7 (14.89%)	105 (2.24%)	0.000069	**0.002337**
25	GO:0042445	hormone metabolic process	4 (8.51%)	27 (0.57%)	0.000131	**0.004253**
26	GO:0060973	cell migration involved in heart development	3 (6.38%)	12 (0.26%)	0.000194	**0.006077**
27	GO:0048232	male gamete generation	6 (12.77%)	89 (1.9%)	0.000225	**0.006783**
28	GO:0001508	action potential	4 (8.51%)	33 (0.7%)	0.000291	**0.008206**
29	GO:0010817	regulation of hormone levels	5 (10.64%)	60 (1.28%)	0.000292	**0.008206**
30	GO:0055117	regulation of cardiac muscle contraction	3 (6.38%)	15 (0.32%)	0.000393	**0.010665**
31	GO:0042592	homeostatic process	13 (27.66%)	462 (9.84%)	0.000418	**0.010835**
32	GO:0015992	proton transport	5 (10.64%)	65 (1.38%)	0.000426	**0.010835**
33	GO:0006936	muscle contraction	7 (14.89%)	143 (3.05%)	0.000475	**0.011717**
34	GO:0042391	regulation of membrane potential	5 (10.64%)	71 (1.51%)	0.000642	**0.014954**
35	GO:0019725	cellular homeostasis	9 (19.15%)	248 (5.28%)	0.000643	**0.014954**
36	GO:0009719	response to endogenous stimulus	13 (27.66%)	493 (10.5%)	0.000785	**0.017747**
37	GO:0006818	hydrogen transport	5 (10.64%)	77 (1.64%)	0.000932	**0.020184**
38	GO:1902307	positive regulation of sodium ion transmembrane transport	2 (4.26%)	5 (0.11%)	0.000962	**0.020184**
39	GO:0006875	cellular metal ion homeostasis	7 (14.89%)	161 (3.43%)	0.000967	**0.020184**
40	GO:0030003	cellular cation homeostasis	7 (14.89%)	167 (3.56%)	0.001200	**0.024415**
41	GO:0060048	cardiac muscle contraction	3 (6.38%)	22 (0.47%)	0.001267	**0.025145**
42	GO:1902600	hydrogen ion transmembrane transport	3 (6.38%)	23 (0.49%)	0.001446	**0.027914**
43	GO:0006873	cellular ion homeostasis	7 (14.89%)	173 (3.68%)	0.001475	**0.027914**
44	GO:0003012	muscle system process	8 (17.02%)	225 (4.79%)	0.001524	**0.028196**
45	GO:0031214	biomineral tissue development	3 (6.38%)	24 (0.51%)	0.001641	**0.029691**

46	GO:0043062	extracellular structure organization	7 (14.89%)	177 (3.77%)	0.001684	**0.029793**
47	GO:0006942	regulation of striated muscle contraction	3 (6.38%)	25 (0.53%)	0.001852	**0.032078**
48	GO:0007276	gamete generation	6 (12.77%)	134 (2.85%)	0.001980	**0.032468**
49	GO:0043268	positive regulation of potassium ion transport	2 (4.26%)	7 (0.15%)	0.001994	**0.032468**
50	GO:1901381	positive regulation of potassium ion transmembrane transport	2 (4.26%)	7 (0.15%)	0.001994	**0.032468**
51	GO:0009725	response to hormone	10 (21.28%)	355 (7.56%)	0.002212	**0.035300**
52	GO:0034220	ion transmembrane transport	6 (12.77%)	138 (2.94%)	0.002300	**0.035455**
53	GO:0055082	cellular chemical homeostasis	7 (14.89%)	187 (3.98%)	0.002308	**0.035455**
54	GO:0055065	metal ion homeostasis	7 (14.89%)	190 (4.05%)	0.002527	**0.038096**
55	GO:1901021	positive regulation of calcium ion transmembrane transporter activity	2 (4.26%)	8 (0.17%)	0.002642	**0.038407**
56	GO:1904427	positive regulation of calcium ion transmembrane transport	2 (4.26%)	8 (0.17%)	0.002642	**0.038407**
57	GO:0010765	positive regulation of sodium ion transport	2 (4.26%)	9 (0.19%)	0.003376	**0.047374**
58	GO:1901379	regulation of potassium ion transmembrane transport	2 (4.26%)	9 (0.19%)	0.003376	**0.047374**
59	GO:0055085	transmembrane transport	6 (12.77%)	151 (3.22%)	0.003614	**0.049865**
60	GO:0007423	sensory organ development	6 (12.77%)	152 (3.24%)	0.003735	0.050291
61	GO:0055080	cation homeostasis	7 (14.89%)	204 (4.34%)	0.003769	0.050291
62	GO:0048609	multicellular organismal reproductive process	6 (12.77%)	155 (3.3%)	0.004114	0.054007
63	GO:0010669	epithelial structure maintenance	2 (4.26%)	10 (0.21%)	0.004193	0.054170
64	GO:0098771	inorganic ion homeostasis	7 (14.89%)	210 (4.47%)	0.004425	0.056282
65	GO:0032504	multicellular organism reproduction	6 (12.77%)	160 (3.41%)	0.004808	0.060076
66	GO:0006812	cation transport	8 (17.02%)	271 (5.77%)	0.004871	0.060076
67	GO:0019953	sexual reproduction	6 (12.77%)	165 (3.51%)	0.005586	0.066822
68	GO:0050801	ion homeostasis	7 (14.89%)	220 (4.68%)	0.005709	0.066822

69	GO:0009799	specification of symmetry	3 (6.38%)	37 (0.79%)	0.005753	0.066822
70	GO:0009855	determination of bilateral symmetry	3 (6.38%)	37 (0.79%)	0.005753	0.066822
71	GO:0015669	gas transport	2 (4.26%)	12 (0.26%)	0.006071	0.066822
72	GO:1902305	regulation of sodium ion transmembrane transport	2 (4.26%)	12 (0.26%)	0.006071	0.066822
73	GO:1904064	positive regulation of cation transmembrane transport	2 (4.26%)	12 (0.26%)	0.006071	0.066822
74	GO:0009612	response to mechanical stimulus	4 (8.51%)	74 (1.58%)	0.006075	0.066822
75	GO:0008016	regulation of heart contraction	4 (8.51%)	75 (1.6%)	0.006370	0.069141
76	GO:0051928	positive regulation of calcium ion transport	2 (4.26%)	13 (0.28%)	0.007129	0.075369
77	GO:0060343	trabecula formation	2 (4.26%)	13 (0.28%)	0.007129	0.075369
78	GO:0048878	chemical homeostasis	8 (17.02%)	292 (6.22%)	0.007606	0.079094
79	GO:0050953	sensory perception of light stimulus	3 (6.38%)	41 (0.87%)	0.007676	0.079094
80	GO:0060047	heart contraction	4 (8.51%)	81 (1.72%)	0.008348	0.084938
81	GO:0019216	regulation of lipid metabolic process	4 (8.51%)	83 (1.77%)	0.009087	0.091319
82	GO:0001974	blood vessel remodeling	1 (2.13%)	1 (0.02%)	0.010009	0.096987
83	GO:0010507	negative regulation of autophagy	1 (2.13%)	1 (0.02%)	0.010009	0.096987
84	GO:0030540	female genitalia development	1 (2.13%)	1 (0.02%)	0.010009	0.096987
85	GO:0006024	glycosaminoglycan biosynthetic process	2 (4.26%)	16 (0.34%)	0.010761	0.100684
86	GO:0006027	glycosaminoglycan catabolic process	2 (4.26%)	16 (0.34%)	0.010761	0.100684
87	GO:0061383	trabecula morphogenesis	2 (4.26%)	16 (0.34%)	0.010761	0.100684
88	GO:0008202	steroid metabolic process	4 (8.51%)	88 (1.87%)	0.011120	0.102860
89	GO:0003008	system process	11 (23.4%)	521 (11.09%)	0.011882	0.108369
90	GO:0044702	single organism reproductive process	7 (14.89%)	253 (5.39%)	0.011982	0.108369
91	GO:0044703	multi-organism reproductive process	6 (12.77%)	196 (4.17%)	0.012614	0.110441
92	GO:0048870	cell motility	10 (21.28%)	454 (9.67%)	0.012618	0.110441

93	GO:0051674	localization of cell	10 (21.28%)	454 (9.67%)	0.012618	0.110441
94	GO:0006937	regulation of muscle contraction	3 (6.38%)	50 (1.06%)	0.013257	0.114798
95	GO:0016477	cell migration	9 (19.15%)	392 (8.35%)	0.014093	0.120128
96	GO:0006629	lipid metabolic process	10 (21.28%)	462 (9.84%)	0.014167	0.120128
97	GO:0030001	metal ion transport	6 (12.77%)	203 (4.32%)	0.014812	0.123029
98	GO:0048598	embryonic morphogenesis	6 (12.77%)	203 (4.32%)	0.014812	0.123029
99	GO:0001701	in utero embryonic development	2 (4.26%)	21 (0.45%)	0.018244	0.142390
100	GO:0006026	aminoglycan catabolic process	2 (4.26%)	21 (0.45%)	0.018244	0.142390
101	GO:0042476	odontogenesis	2 (4.26%)	21 (0.45%)	0.018244	0.142390
102	GO:1901019	regulation of calcium ion transmembrane transporter activity	2 (4.26%)	21 (0.45%)	0.018244	0.142390
103	GO:1903522	regulation of blood circulation	4 (8.51%)	104 (2.21%)	0.019526	0.142390
104	GO:0007622	rhythmic behavior	1 (2.13%)	2 (0.04%)	0.019919	0.142390
105	GO:0022410	circadian sleep/wake cycle process	1 (2.13%)	2 (0.04%)	0.019919	0.142390
106	GO:0032594	protein transport within lipid bilayer	1 (2.13%)	2 (0.04%)	0.019919	0.142390
107	GO:0032596	protein transport into membrane raft	1 (2.13%)	2 (0.04%)	0.019919	0.142390
108	GO:0042635	positive regulation of hair cycle	1 (2.13%)	2 (0.04%)	0.019919	0.142390
109	GO:0042745	circadian sleep/wake cycle	1 (2.13%)	2 (0.04%)	0.019919	0.142390
110	GO:0048512	circadian behavior	1 (2.13%)	2 (0.04%)	0.019919	0.142390
111	GO:0090136	epithelial cell-cell adhesion	1 (2.13%)	2 (0.04%)	0.019919	0.142390
112	GO:1903044	protein localization to membrane raft	1 (2.13%)	2 (0.04%)	0.019919	0.142390
113	GO:0006023	aminoglycan biosynthetic process	2 (4.26%)	22 (0.47%)	0.019942	0.142390
114	GO:0043266	regulation of potassium ion transport	2 (4.26%)	22 (0.47%)	0.019942	0.142390
115	GO:0019932	second-messenger-mediated signaling	3 (6.38%)	60 (1.28%)	0.021594	0.152850
116	GO:0032870	cellular response to hormone stimulus	4 (8.51%)	108 (2.3%)	0.022109	0.155142
117	GO:0010506	regulation of autophagy	3 (6.38%)	61 (1.3%)	0.022555	0.156918
118	GO:0032414	positive regulation of ion transmembrane transporter activity	2 (4.26%)	24 (0.51%)	0.023527	0.161007
119	GO:0030198	extracellular matrix organization	3 (6.38%)	62 (1.32%)	0.023538	0.161007

120	GO:0071495	cellular response to endogenous stimulus	5 (10.64%)	167 (3.56%)	0.024607	0.166918
121	GO:0031330	negative regulation of cellular catabolic process	2 (4.26%)	25 (0.53%)	0.025412	0.169550
122	GO:1903169	regulation of calcium ion transmembrane transport	2 (4.26%)	25 (0.53%)	0.025412	0.169550
123	GO:0033273	response to vitamin	2 (4.26%)	26 (0.55%)	0.027356	0.179579
124	GO:0070588	calcium ion transmembrane transport	2 (4.26%)	26 (0.55%)	0.027356	0.179579
125	GO:0006914	autophagy	3 (6.38%)	66 (1.41%)	0.027703	0.180401
126	GO:0070849	response to epidermal growth factor	2 (4.26%)	27 (0.57%)	0.029359	0.184749
127	GO:0006020	inositol metabolic process	1 (2.13%)	3 (0.06%)	0.029732	0.184749
128	GO:0010715	regulation of extracellular matrix disassembly	1 (2.13%)	3 (0.06%)	0.029732	0.184749
129	GO:0018199	peptidyl-glutamine modification	1 (2.13%)	3 (0.06%)	0.029732	0.184749
130	GO:0042483	negative regulation of odontogenesis	1 (2.13%)	3 (0.06%)	0.029732	0.184749
131	GO:1903053	regulation of extracellular matrix organization	1 (2.13%)	3 (0.06%)	0.029732	0.184749
132	GO:0060249	anatomical structure homeostasis	3 (6.38%)	69 (1.47%)	0.031069	0.191591
133	GO:0042743	hydrogen peroxide metabolic process	2 (4.26%)	28 (0.6%)	0.031418	0.192289
134	GO:0001936	regulation of endothelial cell proliferation	2 (4.26%)	29 (0.62%)	0.033534	0.202195
135	GO:0002028	regulation of sodium ion transport	2 (4.26%)	29 (0.62%)	0.033534	0.202195
136	GO:0007417	central nervous system development	5 (10.64%)	183 (3.9%)	0.034728	0.207860
137	GO:0001935	endothelial cell proliferation	2 (4.26%)	30 (0.64%)	0.035703	0.210106
138	GO:0032411	positive regulation of transporter activity	2 (4.26%)	30 (0.64%)	0.035703	0.210106
139	GO:0043627	response to estrogen	3 (6.38%)	73 (1.55%)	0.035878	0.210106
140	GO:0051239	regulation of multicellular organismal process	11 (23.4%)	615 (13.1%)	0.036731	0.213360
141	GO:0022414	reproductive process	7 (14.89%)	318 (6.77%)	0.036958	0.213360
142	GO:0019722	calcium-mediated signaling	2 (4.26%)	31 (0.66%)	0.037926	0.215888
143	GO:0034767	positive regulation of ion transmembrane transport	2 (4.26%)	31 (0.66%)	0.037926	0.215888
144	GO:0000003	reproduction	7 (14.89%)	321 (6.84%)	0.038608	0.216714

145	GO:0006811	ion transport	8 (17.02%)	393 (8.37%)	0.039016	0.216714
146	GO:0007215	glutamate receptor signaling pathway	1 (2.13%)	4 (0.09%)	0.039449	0.216714
147	GO:0007601	visual perception	1 (2.13%)	4 (0.09%)	0.039449	0.216714
148	GO:0036072	direct ossification	1 (2.13%)	4 (0.09%)	0.039449	0.216714
149	GO:0008406	gonad development	2 (4.26%)	32 (0.68%)	0.040201	0.216714
150	GO:0030203	glycosaminoglycan metabolic process	2 (4.26%)	32 (0.68%)	0.040201	0.216714
151	GO:0045137	development of primary sexual characteristics	2 (4.26%)	32 (0.68%)	0.040201	0.216714
152	GO:0043462	regulation of ATPase activity	2 (4.26%)	33 (0.7%)	0.042527	0.227742
153	GO:0000302	response to reactive oxygen species	3 (6.38%)	79 (1.68%)	0.043772	0.232879
154	GO:0009790	embryo development	8 (17.02%)	406 (8.65%)	0.045965	0.237762
155	GO:0006979	response to oxidative stress	4 (8.51%)	136 (2.9%)	0.045968	0.237762
156	GO:0072358	cardiovascular system development	6 (12.77%)	264 (5.62%)	0.046147	0.237762
157	GO:0072359	circulatory system development	6 (12.77%)	264 (5.62%)	0.046147	0.237762
158	GO:0048545	response to steroid hormone	5 (10.64%)	199 (4.24%)	0.047139	0.237762
159	GO:0009895	negative regulation of catabolic process	2 (4.26%)	35 (0.75%)	0.047325	0.237762
160	GO:0034764	positive regulation of transmembrane transport	2 (4.26%)	35 (0.75%)	0.047325	0.237762
161	GO:0002639	positive regulation of immunoglobulin production	1 (2.13%)	5 (0.11%)	0.049071	0.237762
162	GO:0018149	peptide cross-linking	1 (2.13%)	5 (0.11%)	0.049071	0.237762
163	GO:0019042	viral latency	1 (2.13%)	5 (0.11%)	0.049071	0.237762
164	GO:0019043	establishment of viral latency	1 (2.13%)	5 (0.11%)	0.049071	0.237762
165	GO:0022617	extracellular matrix disassembly	1 (2.13%)	5 (0.11%)	0.049071	0.237762
166	GO:0035728	response to hepatocyte growth factor	1 (2.13%)	5 (0.11%)	0.049071	0.237762
167	GO:0042481	regulation of odontogenesis	1 (2.13%)	5 (0.11%)	0.049071	0.237762
168	GO:0042634	regulation of hair cycle	1 (2.13%)	5 (0.11%)	0.049071	0.237762

B. Molecular Function of upregulated genes

#	GO ID	Description	Freshwater vs Salinity UP (39)	All (4619)	*p-value*	*p-adjust*
1	GO:0008556	potassium-transporting ATPase activity	6 (15.38%)	20 (0.43%)	0.000000	**0.000001**
2	GO:0015079	potassium ion transmembrane transporter activity	6 (15.38%)	21 (0.45%)	0.000000	**0.000001**
3	GO:0015662	ATPase activity, coupled to transmembrane	6 (15.38%)	32 (0.69%)	0.000000	**0.000008**

		movement of ions, phosphorylative mechanism				
4	GO:0019829	cation-transporting ATPase activity	6 (15.38%)	37 (0.8%)	0.000000	**0.000015**
5	GO:0042625	ATPase coupled ion transmembrane transporter activity	6 (15.38%)	41 (0.89%)	0.000001	**0.000023**
6	GO:0016820	hydrolase activity, acting on acid anhydrides, catalyzing transmembrane movement of substances	6 (15.38%)	44 (0.95%)	0.000001	**0.000023**
7	GO:0042626	ATPase activity, coupled to transmembrane movement of substances	6 (15.38%)	44 (0.95%)	0.000001	**0.000023**
8	GO:0043492	ATPase activity, coupled to movement of substances	6 (15.38%)	44 (0.95%)	0.000001	**0.000023**
9	GO:0046873	metal ion transmembrane transporter activity	6 (15.38%)	47 (1.02%)	0.000002	**0.000030**
10	GO:0015399	primary active transmembrane transporter activity	6 (15.38%)	50 (1.08%)	0.000003	**0.000036**
11	GO:0015405	P-P-bond-hydrolysis-driven transmembrane transporter activity	6 (15.38%)	50 (1.08%)	0.000003	**0.000036**
12	GO:0015077	monovalent inorganic cation transmembrane transporter activity	6 (15.38%)	59 (1.28%)	0.000008	**0.000087**
13	GO:0031420	Alkaline metal ion binding	4 (10.26%)	17 (0.37%)	0.000010	**0.000098**
14	GO:0022804	active transmembrane transporter activity	6 (15.38%)	72 (1.56%)	0.000025	**0.000240**
15	GO:0022892	substrate-specific transporter activity	10 (25.64%)	254 (5.5%)	0.000033	**0.000285**
16	GO:0005496	steroid binding	4 (10.26%)	23 (0.5%)	0.000034	**0.000285**
17	GO:0022890	inorganic cation transmembrane transporter activity	6 (15.38%)	79 (1.71%)	0.000043	**0.000337**
18	GO:0005215	transporter activity	10 (25.64%)	279 (6.04%)	0.000073	**0.000540**
19	GO:0008324	cation transmembrane transporter activity	6 (15.38%)	110 (2.38%)	0.000274	**0.001912**
20	GO:0042623	ATPase activity, coupled	6 (15.38%)	111 (2.4%)	0.000288	**0.001912**
21	GO:0016887	ATPase activity	6 (15.38%)	129 (2.79%)	0.000646	**0.004093**
22	GO:0008289	lipid binding	7 (17.95%)	195 (4.22%)	0.001044	**0.006313**
23	GO:0015075	ion transmembrane transporter activity	6 (15.38%)	153 (3.31%)	0.001580	**0.009136**
24	GO:0042578	phosphoric ester hydrolase activity	6 (15.38%)	162 (3.51%)	0.002117	**0.011734**
25	GO:0005501	retinoid binding	2 (5.13%)	10 (0.22%)	0.002996	**0.015324**
26	GO:0019840	isoprenoid binding	2 (5.13%)	10 (0.22%)	0.002996	**0.015324**
27	GO:0022891	substrate-specific transmembrane transporter activity	6 (15.38%)	179 (3.88%)	0.003500	**0.017238**
28	GO:0022857	transmembrane transporter activity	6 (15.38%)	196 (4.24%)	0.005469	**0.025978**
29	GO:0004866	endopeptidase inhibitor activity	2 (5.13%)	15 (0.32%)	0.006806	**0.030173**
30	GO:0030414	peptidase inhibitor activity	2 (5.13%)	15 (0.32%)	0.006806	**0.030173**
31	GO:0016853	isomerase activity	3 (7.69%)	49 (1.06%)	0.007843	**0.033649**
32	GO:0016872	intramolecular lyase activity	1 (2.56%)	1 (0.02%)	0.008443	**0.034029**
33	GO:0016972	thiol oxidase activity	1 (2.56%)	1 (0.02%)	0.008443	**0.034029**
34	GO:0004601	peroxidase activity	2 (5.13%)	22 (0.48%)	0.014426	0.056433
35	GO:0016684	oxidoreductase activity, acting on peroxide as	2 (5.13%)	23 (0.5%)	0.015717	0.059724

		acceptor				
36	GO:0009055	electron carrier activity	2 (5.13%)	25 (0.54%)	0.018440	0.068126
37	GO:0032403	protein complex binding	4 (10.26%)	123 (2.66%)	0.019186	0.068968
38	GO:0023023	MHC protein complex binding	2 (5.13%)	26 (0.56%)	0.019871	0.069550
39	GO:0016860	intramolecular oxidoreductase activity	2 (5.13%)	27 (0.58%)	0.021348	0.072802
40	GO:0061135	endopeptidase regulator activity	2 (5.13%)	28 (0.61%)	0.022869	0.076039
41	GO:0005391	sodium:potassium-exchanging ATPase activity	1 (2.56%)	3 (0.06%)	0.025122	0.081494
42	GO:0016209	antioxidant activity	2 (5.13%)	33 (0.71%)	0.031112	0.098521
43	GO:0016788	hydrolase activity, acting on ester bonds	6 (15.38%)	289 (6.26%)	0.032387	0.098594
44	GO:0004869	cysteine-type endopeptidase inhibitor activity	1 (2.56%)	4 (0.09%)	0.033359	0.098594
45	GO:0016670	oxidoreductase activity, acting on a sulfur group of donors, oxygen as acceptor	1 (2.56%)	4 (0.09%)	0.033359	0.098594
46	GO:0003823	antigen binding	2 (5.13%)	35 (0.76%)	0.034692	0.100306
47	GO:0046906	tetrapyrrole binding	2 (5.13%)	37 (0.8%)	0.038425	0.108736
48	GO:0043236	laminin binding	1 (2.56%)	5 (0.11%)	0.041528	0.112533
49	GO:0050840	extracellular matrix binding	1 (2.56%)	5 (0.11%)	0.041528	0.112533
50	GO:0061134	peptidase regulator activity	2 (5.13%)	39 (0.84%)	0.042306	0.112533
51	GO:0017111	nucleoside-triphosphatase activity	7 (17.95%)	404 (8.75%)	0.049417	0.126936
52	GO:0016864	intramolecular oxidoreductase activity, transposing S-S bonds	1 (2.56%)	6 (0.13%)	0.049629	0.126936

C. Cellular Component of upregulated genes

#	GO ID	Description	Freshwater vs Salinity UP (46)	All (4741)	*p-value*	*p-adjust*
1	GO:0031012	extracellular matrix	7 (15.22%)	145 (3.06%)	0.000426	**0.029859**
2	GO:0044291	cell-cell contact zone	4 (8.7%)	43 (0.91%)	0.000726	**0.029859**
3	GO:0005576	extracellular region	12 (26.09%)	445 (9.39%)	0.000807	**0.029859**
4	GO:0044421	extracellular region part	11 (23.91%)	427 (9.01%)	0.002032	0.056385
5	GO:0031988	membrane-bounded vesicle	26 (56.52%)	1701 (35.88%)	0.003268	0.072541
6	GO:0031982	vesicle	26 (56.52%)	1739 (36.68%)	0.004627	0.075467
7	GO:0098794	postsynapse	3 (6.52%)	36 (0.76%)	0.004879	0.075467
8	GO:0048770	pigment granule	4 (8.7%)	74 (1.56%)	0.005439	0.075467
9	GO:0044420	extracellular matrix component	3 (6.52%)	68 (1.43%)	0.027617	0.248379
10	GO:0030175	filopodium	1 (2.17%)	3 (0.06%)	0.028832	0.248379
11	GO:0005886	plasma membrane	9 (19.57%)	462 (9.74%)	0.030792	0.248379
12	GO:0005882	intermediate filament	2 (4.35%)	30 (0.63%)	0.033725	0.248379
13	GO:0045111	intermediate filament cytoskeleton	2 (4.35%)	30 (0.63%)	0.033725	0.248379
14	GO:0014069	postsynaptic density	2 (4.35%)	31 (0.65%)	0.035832	0.248379
15	GO:0060076	excitatory synapse	2 (4.35%)	31 (0.65%)	0.035832	0.248379
16	GO:0099572	postsynaptic specialization	2 (4.35%)	31 (0.65%)	0.035832	0.248379
17	GO:0045202	synapse	4 (8.7%)	132 (2.78%)	0.038040	0.248379
18	GO:0071944	cell periphery	9 (19.57%)	488 (10.29%)	0.041801	0.257775

19	GO:0005911	cell-cell junction	4 (8.7%)	140 (2.95%)	0.045648	0.264182
20	GO:0070701	mucus layer	1 (2.17%)	5 (0.11%)	0.047600	0.264182
21	GO:0043197	dendritic spine	1 (2.17%)	6 (0.13%)	0.056851	0.293110

D. Biological Process of downregulated genes

#	GO ID	Description	Freshwater vs Salinity DOWN (53)	All (4696)	*p-value*	*p-adjust*
1	GO:0009268	response to pH	2 (3.77%)	11 (0.23%)	0.006441	0.954481
2	GO:0045646	regulation of erythrocyte differentiation	1 (1.89%)	1 (0.02%)	0.011286	0.954481
3	GO:0061582	intestinal epithelial cell migration	1 (1.89%)	1 (0.02%)	0.011286	0.954481
4	GO:0051707	response to other organism	8 (15.09%)	288 (6.13%)	0.014379	0.954481
5	GO:0043207	response to external biotic stimulus	8 (15.09%)	293 (6.24%)	0.015827	0.954481
6	GO:0009607	response to biotic stimulus	8 (15.09%)	297 (6.32%)	0.017060	0.954481
7	GO:0032846	positive regulation of homeostatic process	2 (3.77%)	20 (0.43%)	0.020855	0.954481
8	GO:0000460	maturation of 5.8S rRNA	1 (1.89%)	2 (0.04%)	0.022447	0.954481
9	GO:0000470	maturation of LSU-rRNA	1 (1.89%)	2 (0.04%)	0.022447	0.954481
10	GO:0042273	ribosomal large subunit biogenesis	1 (1.89%)	2 (0.04%)	0.022447	0.954481
11	GO:0070391	response to lipoteichoic acid	1 (1.89%)	2 (0.04%)	0.022447	0.954481
12	GO:0002221	pattern recognition receptor signaling pathway	3 (5.66%)	54 (1.15%)	0.022465	0.954481
13	GO:0006664	glycolipid metabolic process	2 (3.77%)	21 (0.45%)	0.022886	0.954481
14	GO:0045069	regulation of viral genome replication	2 (3.77%)	21 (0.45%)	0.022886	0.954481
15	GO:1903509	liposaccharide metabolic process	2 (3.77%)	21 (0.45%)	0.022886	0.954481
16	GO:0009410	response to xenobiotic stimulus	2 (3.77%)	22 (0.47%)	0.024994	0.954481
17	GO:0031349	positive regulation of defense response	5 (9.43%)	150 (3.19%)	0.026045	0.954481
18	GO:0019079	viral genome replication	2 (3.77%)	24 (0.51%)	0.029439	0.954481
19	GO:0006400	tRNA modification	1 (1.89%)	3 (0.06%)	0.033485	0.954481
20	GO:0006566	threonine metabolic process	1 (1.89%)	3 (0.06%)	0.033485	0.954481
21	GO:0039531	regulation of viral-induced cytoplasmic pattern recognition receptor signaling pathway	1 (1.89%)	3 (0.06%)	0.033485	0.954481
22	GO:0039532	negative regulation of viral-induced cytoplasmic pattern recognition receptor signaling pathway	1 (1.89%)	3 (0.06%)	0.033485	0.954481
23	GO:0061337	cardiac conduction	1 (1.89%)	3 (0.06%)	0.033485	0.954481
24	GO:0086065	cell communication involved in cardiac conduction	1 (1.89%)	3 (0.06%)	0.033485	0.954481
25	GO:0009451	RNA modification	2 (3.77%)	27 (0.57%)	0.036646	0.954481

26	GO:0002758	innate immune response-activating signal transduction	4 (7.55%)	119 (2.53%)	0.044292	0.954481
27	GO:0035637	multicellular organismal signaling	1 (1.89%)	4 (0.09%)	0.044400	0.954481
28	GO:0051931	regulation of sensory perception	1 (1.89%)	4 (0.09%)	0.044400	0.954481
29	GO:0086010	membrane depolarization during action potential	1 (1.89%)	4 (0.09%)	0.044400	0.954481
30	GO:0002218	activation of innate immune response	4 (7.55%)	120 (2.56%)	0.045447	0.954481
31	GO:0045088	regulation of innate immune response	4 (7.55%)	122 (2.6%)	0.047807	0.954481
32	GO:0045089	positive regulation of innate immune response	4 (7.55%)	122 (2.6%)	0.047807	0.954481

E. Molecular Function of downregulated genes

#	GO ID	Description	Freshwater vs Salinity DOWN (50)	All (4619)	***p-value***	***p-adjust***
1	GO:0070011	peptidase activity, acting on L-amino acid peptides	8 (16%)	185 (4.01%)	0.000720	0.068855
2	GO:0008233	peptidase activity	8 (16%)	189 (4.09%)	0.000830	0.068855
3	GO:0008376	acetylgalactosaminyltransferase activity	2 (4%)	6 (0.13%)	0.001676	0.092720
4	GO:0016787	hydrolase activity	20 (40%)	995 (21.54%)	0.002287	0.094927
5	GO:0005248	voltage-gated sodium channel activity	1 (2%)	1 (0.02%)	0.010825	0.138225
6	GO:0005272	sodium channel activity	1 (2%)	1 (0.02%)	0.010825	0.138225
7	GO:0008329	signaling pattern recognition receptor activity	1 (2%)	1 (0.02%)	0.010825	0.138225
8	GO:0016297	acyl-[acyl-carrier-protein] hydrolase activity	1 (2%)	1 (0.02%)	0.010825	0.138225
9	GO:0019171	3-hydroxyacyl-[acyl-carrier-protein] dehydratase activity	1 (2%)	1 (0.02%)	0.010825	0.138225
10	GO:0022843	voltage-gated cation channel activity	1 (2%)	1 (0.02%)	0.010825	0.138225
11	GO:0032453	histone demethylase activity (H3-K4 specific)	1 (2%)	1 (0.02%)	0.010825	0.138225
12	GO:0038187	pattern recognition receptor activity	1 (2%)	1 (0.02%)	0.010825	0.138225
13	GO:0043425	bHLH transcription factor binding	1 (2%)	1 (0.02%)	0.010825	0.138225
14	GO:0001948	glycoprotein binding	2 (4%)	17 (0.37%)	0.014081	0.166957
15	GO:0016728	oxidoreductase activity, acting on CH or CH2 groups, disulfide as acceptor	1 (2%)	2 (0.04%)	0.021535	0.238319
16	GO:0008194	UDP-glycosyltransferase	2 (4%)	23 (0.5%)	0.025137	0.254708

		activity				
17	GO:0004175	endopeptidase activity	4 (8%)	105 (2.27%)	0.026085	0.254708
18	GO:0008484	sulfuric ester hydrolase activity	1 (2%)	3 (0.06%)	0.032131	0.266689
19	GO:0016725	oxidoreductase activity, acting on CH or CH2 groups	1 (2%)	3 (0.06%)	0.032131	0.266689
20	GO:0032452	histone demethylase activity	1 (2%)	3 (0.06%)	0.032131	0.266689

F. Cellular Component of downregulated genes

#	GO ID	Description	Freshwater vs Salinity DOWN (44)	All (4741)	*p-value*	*p-adjust*
1	GO:0000323	lytic vacuole	6 (13.64%)	114 (2.4%)	0.000567	**0.044831**
2	GO:0005773	vacuole	6 (13.64%)	195 (4.11%)	0.008583	0.339013
3	GO:0030684	preribosome	1 (2.27%)	4 (0.08%)	0.036621	0.906154

Table S4. GO Enrichment of Salinity-Alkalinity upregulated genes

A. Biological Process

#	GO ID	Description	Freshwater vs Salinity-Alkalinity UP (39)	All (4696)	*p-value*	*p-adjust*
1	GO:0010107	potassium ion import	6 (15.38%)	12 (0.26%)	0.000000	**0.000000**
2	GO:0071436	sodium ion export	6 (15.38%)	15 (0.32%)	0.000000	**0.000000**
3	GO:0031944	negative regulation of glucocorticoid metabolic process	5 (12.82%)	9 (0.19%)	0.000000	**0.000001**
4	GO:0032351	negative regulation of hormone metabolic process	5 (12.82%)	9 (0.19%)	0.000000	**0.000001**
5	GO:0071804	cellular potassium ion transport	6 (15.38%)	19 (0.4%)	0.000000	**0.000001**
6	GO:0071805	potassium ion transmembrane transport	6 (15.38%)	19 (0.4%)	0.000000	**0.000001**
7	GO:0008211	glucocorticoid metabolic process	5 (12.82%)	10 (0.21%)	0.000000	**0.000001**
8	GO:0031943	regulation of glucocorticoid metabolic process	5 (12.82%)	10 (0.21%)	0.000000	**0.000001**
9	GO:0045939	negative regulation of steroid metabolic process	5 (12.82%)	10 (0.21%)	0.000000	**0.000001**
10	GO:0032350	regulation of hormone metabolic process	5 (12.82%)	11 (0.23%)	0.000000	**0.000001**
11	GO:0045833	negative regulation of lipid metabolic process	5 (12.82%)	12 (0.26%)	0.000000	**0.000002**
12	GO:0010644	cell communication by electrical coupling	6 (15.38%)	24 (0.51%)	0.000000	**0.000002**
13	GO:0035725	sodium ion transmembrane transport	6 (15.38%)	26 (0.55%)	0.000000	**0.000003**
14	GO:0086011	membrane repolarization during action potential	5 (12.82%)	14 (0.3%)	0.000000	**0.000004**
15	GO:0006813	potassium ion transport	6 (15.38%)	35 (0.75%)	0.000000	**0.000017**
16	GO:0086009	membrane repolarization	5 (12.82%)	19 (0.4%)	0.000000	**0.000018**
17	GO:0019218	regulation of steroid metabolic process	5 (12.82%)	23 (0.49%)	0.000001	**0.000047**
18	GO:0042391	regulation of membrane potential	7 (17.95%)	71 (1.51%)	0.000001	**0.000063**
19	GO:0006942	regulation of striated muscle contraction	5 (12.82%)	25 (0.53%)	0.000001	**0.000063**
20	GO:0090075	relaxation of muscle	5 (12.82%)	25 (0.53%)	0.000001	**0.000063**
21	GO:0060973	cell migration involved in heart development	4 (10.26%)	12 (0.26%)	0.000002	**0.000080**
22	GO:0042445	hormone metabolic process	5 (12.82%)	27 (0.57%)	0.000002	**0.000085**
23	GO:0019216	regulation of lipid metabolic process	7 (17.95%)	83 (1.77%)	0.000004	**0.000155**
24	GO:0032504	multicellular organism reproduction	9 (23.08%)	160 (3.41%)	0.000004	**0.000159**
25	GO:0055117	regulation of cardiac muscle contraction	4 (10.26%)	15 (0.32%)	0.000005	**0.000182**
26	GO:0006814	sodium ion transport	6 (15.38%)	57 (1.21%)	0.000006	**0.000197**
27	GO:0001508	action potential	5 (12.82%)	33 (0.7%)	0.000006	**0.000197**
28	GO:0048232	male gamete generation	7 (17.95%)	89 (1.9%)	0.000007	**0.000204**
29	GO:0010817	regulation of hormone levels	6 (15.38%)	60 (1.28%)	0.000008	**0.000240**
30	GO:0098655	cation transmembrane transport	6 (15.38%)	66 (1.41%)	0.000014	**0.000380**
31	GO:0098660	inorganic ion transmembrane transport	6 (15.38%)	66 (1.41%)	0.000014	**0.000380**

32	GO:0098662	inorganic cation transmembrane transport	6 (15.38%)	66 (1.41%)	0.000014	**0.000380**
33	GO:0006941	striated muscle contraction	7 (17.95%)	105 (2.24%)	0.000020	**0.000518**
34	GO:0060048	cardiac muscle contraction	4 (10.26%)	22 (0.47%)	0.000027	**0.000685**
35	GO:0044702	single organism reproductive process	10 (25.64%)	253 (5.39%)	0.000027	**0.000685**
36	GO:0030001	metal ion transport	9 (23.08%)	203 (4.32%)	0.000030	**0.000717**
37	GO:0048609	multicellular organismal reproductive process	8 (20.51%)	155 (3.3%)	0.000030	**0.000717**
38	GO:1902600	hydrogen ion transmembrane transport	4 (10.26%)	23 (0.49%)	0.000032	**0.000741**
39	GO:0006875	cellular metal ion homeostasis	8 (20.51%)	161 (3.43%)	0.000040	**0.000895**
40	GO:0006937	regulation of muscle contraction	5 (12.82%)	50 (1.06%)	0.000049	**0.001071**
41	GO:0030003	cellular cation homeostasis	8 (20.51%)	167 (3.56%)	0.000052	**0.001108**
42	GO:0006873	cellular ion homeostasis	8 (20.51%)	173 (3.68%)	0.000067	**0.001392**
43	GO:0007276	gamete generation	7 (17.95%)	134 (2.85%)	0.000094	**0.001909**
44	GO:0009725	response to hormone	11 (28.21%)	355 (7.56%)	0.000096	**0.001909**
45	GO:0009719	response to endogenous stimulus	13 (33.33%)	493 (10.5%)	0.000100	**0.001941**
46	GO:0055082	cellular chemical homeostasis	8 (20.51%)	187 (3.98%)	0.000116	**0.002205**
47	GO:0055065	metal ion homeostasis	8 (20.51%)	190 (4.05%)	0.000129	**0.002413**
48	GO:0006936	muscle contraction	7 (17.95%)	143 (3.05%)	0.000142	**0.002591**
49	GO:0019725	cellular homeostasis	9 (23.08%)	248 (5.28%)	0.000145	**0.002591**
50	GO:0044703	multi-organism reproductive process	8 (20.51%)	196 (4.17%)	0.000161	**0.002819**
51	GO:0022414	reproductive process	10 (25.64%)	318 (6.77%)	0.000191	**0.003278**
52	GO:0000003	reproduction	10 (25.64%)	321 (6.84%)	0.000206	**0.003474**
53	GO:0055080	cation homeostasis	8 (20.51%)	204 (4.34%)	0.000212	**0.003510**
54	GO:0009799	specification of symmetry	4 (10.26%)	37 (0.79%)	0.000220	**0.003510**
55	GO:0009855	determination of bilateral symmetry	4 (10.26%)	37 (0.79%)	0.000220	**0.003510**
56	GO:0032870	cellular response to hormone stimulus	6 (15.38%)	108 (2.3%)	0.000226	**0.003543**
57	GO:0042592	homeostatic process	12 (30.77%)	462 (9.84%)	0.000234	**0.003591**
58	GO:0051239	regulation of multicellular organismal process	14 (35.9%)	615 (13.1%)	0.000243	**0.003669**
59	GO:0098771	inorganic ion homeostasis	8 (20.51%)	210 (4.47%)	0.000259	**0.003851**
60	GO:0006812	cation transport	9 (23.08%)	271 (5.77%)	0.000284	**0.004150**
61	GO:0009612	response to mechanical stimulus	5 (12.82%)	74 (1.58%)	0.000322	**0.004617**
62	GO:0008016	regulation of heart contraction	5 (12.82%)	75 (1.6%)	0.000342	**0.004815**
63	GO:0019953	sexual reproduction	7 (17.95%)	165 (3.51%)	0.000346	**0.004815**
64	GO:0050801	ion homeostasis	8 (20.51%)	220 (4.68%)	0.000357	**0.004880**
65	GO:0071495	cellular response to endogenous stimulus	7 (17.95%)	167 (3.56%)	0.000373	**0.005024**
66	GO:0007215	glutamate receptor signaling pathway	2 (5.13%)	4 (0.09%)	0.000399	**0.005297**
67	GO:0048771	tissue remodeling	3 (7.69%)	19 (0.4%)	0.000468	**0.006122**
68	GO:0060047	heart contraction	5 (12.82%)	81 (1.72%)	0.000490	**0.006292**
69	GO:0048878	chemical homeostasis	9 (23.08%)	292 (6.22%)	0.000496	**0.006292**
70	GO:0006693	prostaglandin metabolic process	3 (7.69%)	20 (0.43%)	0.000548	**0.006854**
71	GO:0006816	calcium ion transport	5 (12.82%)	85 (1.81%)	0.000613	**0.007557**
72	GO:0008202	steroid metabolic process	5 (12.82%)	88 (1.87%)	0.000718	**0.008740**
73	GO:0015672	monovalent inorganic cation transport	6 (15.38%)	137 (2.92%)	0.000815	**0.009786**

74	GO:0051384	response to glucocorticoid	3 (7.69%)	23 (0.49%)	0.000836	**0.009897**
75	GO:0034220	ion transmembrane transport	6 (15.38%)	138 (2.94%)	0.000847	**0.009897**
76	GO:0006629	lipid metabolic process	11 (28.21%)	462 (9.84%)	0.000966	**0.011135**
77	GO:0006811	ion transport	10 (25.64%)	393 (8.37%)	0.001046	**0.011896**
78	GO:0006692	prostanoid metabolic process	3 (7.69%)	25 (0.53%)	0.001074	**0.011906**
79	GO:0034614	cellular response to reactive oxygen species	3 (7.69%)	25 (0.53%)	0.001074	**0.011906**
80	GO:0031960	response to corticosteroid	4 (10.26%)	57 (1.21%)	0.001170	**0.012810**
81	GO:0070838	divalent metal ion transport	5 (12.82%)	99 (2.11%)	0.001227	**0.013108**
82	GO:0072511	divalent inorganic cation transport	5 (12.82%)	99 (2.11%)	0.001227	**0.013108**
83	GO:0090257	regulation of muscle system process	5 (12.82%)	100 (2.13%)	0.001284	**0.013549**
84	GO:0055085	transmembrane transport	6 (15.38%)	151 (3.22%)	0.001357	**0.014148**
85	GO:0010033	response to organic substance	15 (38.46%)	816 (17.38%)	0.001401	**0.014436**
86	GO:1903522	regulation of blood circulation	5 (12.82%)	104 (2.21%)	0.001531	**0.015591**
87	GO:0044057	regulation of system process	6 (15.38%)	159 (3.39%)	0.001770	**0.017750**
88	GO:0010519	negative regulation of phospholipase activity	2 (5.13%)	8 (0.17%)	0.001824	**0.017750**
89	GO:0032717	negative regulation of interleukin-8 production	2 (5.13%)	8 (0.17%)	0.001824	**0.017750**
90	GO:0060192	negative regulation of lipase activity	2 (5.13%)	8 (0.17%)	0.001824	**0.017750**
91	GO:0015992	proton transport	4 (10.26%)	65 (1.38%)	0.001912	**0.018403**
92	GO:0003012	muscle system process	7 (17.95%)	225 (4.79%)	0.002183	**0.020638**
93	GO:0001516	prostaglandin biosynthetic process	2 (5.13%)	9 (0.19%)	0.002332	**0.020638**
94	GO:0031392	regulation of prostaglandin biosynthetic process	2 (5.13%)	9 (0.19%)	0.002332	**0.020638**
95	GO:0042304	regulation of fatty acid biosynthetic process	2 (5.13%)	9 (0.19%)	0.002332	**0.020638**
96	GO:0046456	icosanoid biosynthetic process	2 (5.13%)	9 (0.19%)	0.002332	**0.020638**
97	GO:0046457	prostanoid biosynthetic process	2 (5.13%)	9 (0.19%)	0.002332	**0.020638**
98	GO:1901570	fatty acid derivative biosynthetic process	2 (5.13%)	9 (0.19%)	0.002332	**0.020638**
99	GO:2001279	regulation of unsaturated fatty acid biosynthetic process	2 (5.13%)	9 (0.19%)	0.002332	**0.020638**
100	GO:0007611	learning or memory	3 (7.69%)	35 (0.75%)	0.002885	**0.025022**
101	GO:0050890	cognition	3 (7.69%)	35 (0.75%)	0.002885	**0.025022**
102	GO:0006818	hydrogen transport	4 (10.26%)	77 (1.64%)	0.003558	**0.030557**
103	GO:0002252	immune effector process	6 (15.38%)	186 (3.96%)	0.003903	**0.032847**
104	GO:0006690	icosanoid metabolic process	3 (7.69%)	39 (0.83%)	0.003937	**0.032847**
105	GO:1901568	fatty acid derivative metabolic process	3 (7.69%)	39 (0.83%)	0.003937	**0.032847**
106	GO:0015669	gas transport	2 (5.13%)	12 (0.26%)	0.004209	**0.034012**
107	GO:0032637	interleukin-8 production	2 (5.13%)	12 (0.26%)	0.004209	**0.034012**
108	GO:0032677	regulation of interleukin-8 production	2 (5.13%)	12 (0.26%)	0.004209	**0.034012**
109	GO:0033559	unsaturated fatty acid metabolic process	3 (7.69%)	40 (0.85%)	0.004232	**0.034012**
110	GO:0034599	cellular response to oxidative stress	3 (7.69%)	41 (0.87%)	0.004540	**0.035831**
111	GO:0051924	regulation of calcium ion transport	3 (7.69%)	41 (0.87%)	0.004540	**0.035831**

112	GO:0006979	response to oxidative stress	5 (12.82%)	136 (2.9%)	0.004936	**0.037695**
113	GO:0051928	positive regulation of calcium ion transport	2 (5.13%)	13 (0.28%)	0.004948	**0.037695**
114	GO:1901989	positive regulation of cell cycle phase transition	2 (5.13%)	13 (0.28%)	0.004948	**0.037695**
115	GO:1901992	positive regulation of mitotic cell cycle phase transition	2 (5.13%)	13 (0.28%)	0.004948	**0.037695**
116	GO:0048545	response to steroid hormone	6 (15.38%)	199 (4.24%)	0.005437	**0.041060**
117	GO:0032309	icosanoid secretion	2 (5.13%)	14 (0.3%)	0.005743	**0.042700**
118	GO:0003015	heart process	5 (12.82%)	141 (3%)	0.005752	**0.042700**
119	GO:0048598	embryonic morphogenesis	6 (15.38%)	203 (4.32%)	0.005988	**0.044082**
120	GO:0009628	response to abiotic stimulus	8 (20.51%)	344 (7.33%)	0.006294	**0.045945**
121	GO:0070887	cellular response to chemical stimulus	9 (23.08%)	423 (9.01%)	0.006571	**0.046948**
122	GO:0071715	icosanoid transport	2 (5.13%)	15 (0.32%)	0.006592	**0.046948**
123	GO:1901571	fatty acid derivative transport	2 (5.13%)	15 (0.32%)	0.006592	**0.046948**

B. Molecular Function

#	GO ID	Description	Freshwater vs Salinity-Alkalinity UP (36)	All (4619)	*p-value*	*p-adjust*
1	GO:0008556	potassium-transporting ATPase activity	6 (16.67%)	20 (0.43%)	0.000000	**0.000000**
2	GO:0015079	potassium ion transmembrane transporter activity	6 (16.67%)	21 (0.45%)	0.000000	**0.000000**
3	GO:0015662	ATPase activity, coupled to transmembrane movement of ions, phosphorylative mechanism	6 (16.67%)	32 (0.69%)	0.000000	**0.000004**
4	GO:0031420	Alkaline metal ion binding	5 (13.89%)	17 (0.37%)	0.000000	**0.000004**
5	GO:0019829	cation-transporting ATPase activity	6 (16.67%)	37 (0.8%)	0.000000	**0.000007**
6	GO:0042625	ATPase coupled ion transmembrane transporter activity	6 (16.67%)	41 (0.89%)	0.000001	**0.000010**
7	GO:0005496	steroid binding	5 (13.89%)	23 (0.5%)	0.000001	**0.000010**
8	GO:0016820	hydrolase activity, acting on acid anhydrides, catalyzing transmembrane movement of substances	6 (16.67%)	44 (0.95%)	0.000001	**0.000010**
9	GO:0042626	ATPase activity, coupled to transmembrane movement of substances	6 (16.67%)	44 (0.95%)	0.000001	**0.000010**
10	GO:0043492	ATPase activity, coupled to movement of substances	6 (16.67%)	44 (0.95%)	0.000001	**0.000010**
11	GO:0046873	metal ion transmembrane transporter activity	6 (16.67%)	47 (1.02%)	0.000001	**0.000013**
12	GO:0008289	lipid binding	10 (27.78%)	195 (4.22%)	0.000001	**0.000014**
13	GO:0042623	ATPase activity, coupled	8 (22.22%)	111 (2.4%)	0.000001	**0.000014**
14	GO:0015399	primary active transmembrane transporter activity	6 (16.67%)	50 (1.08%)	0.000002	**0.000014**
15	GO:0015405	P-P-bond-hydrolysis-driven	6 (16.67%)	50 (1.08%)	0.000002	**0.000014**

		transmembrane transporter activity				
16	GO:0016887	ATPase activity	8 (22.22%)	129 (2.79%)	0.000005	**0.000034**
17	GO:0015077	monovalent inorganic cation transmembrane transporter activity	6 (16.67%)	59 (1.28%)	0.000005	**0.000034**
18	GO:0022804	active transmembrane transporter activity	6 (16.67%)	72 (1.56%)	0.000016	**0.000104**
19	GO:0022890	inorganic cation transmembrane transporter activity	6 (16.67%)	79 (1.71%)	0.000027	**0.000169**
20	GO:0022892	substrate-specific transporter activity	9 (25%)	254 (5.5%)	0.000101	**0.000608**
21	GO:0008324	cation transmembrane transporter activity	6 (16.67%)	110 (2.38%)	0.000173	**0.000962**
22	GO:0005391	sodium:potassium-exchanging ATPase activity	2 (5.56%)	3 (0.06%)	0.000176	**0.000962**
23	GO:0005215	transporter activity	9 (25%)	279 (6.04%)	0.000208	**0.001087**
24	GO:0015075	ion transmembrane transporter activity	6 (16.67%)	153 (3.31%)	0.001024	**0.005119**
25	GO:0015081	sodium ion transmembrane transporter activity	2 (5.56%)	7 (0.15%)	0.001210	**0.005752**
26	GO:0061135	endopeptidase regulator activity	3 (8.33%)	28 (0.61%)	0.001246	**0.005752**
27	GO:0042578	phosphoric ester hydrolase activity	6 (16.67%)	162 (3.51%)	0.001379	**0.006128**
28	GO:0004859	phospholipase inhibitor activity	2 (5.56%)	9 (0.19%)	0.002055	**0.008805**
29	GO:0022891	substrate-specific transmembrane transporter activity	6 (16.67%)	179 (3.88%)	0.002300	**0.009518**
30	GO:0005501	retinoid binding	2 (5.56%)	10 (0.22%)	0.002556	**0.009893**
31	GO:0019840	isoprenoid binding	2 (5.56%)	10 (0.22%)	0.002556	**0.009893**
32	GO:0017111	nucleoside-triphosphatase activity	9 (25%)	404 (8.75%)	0.003043	**0.011410**
33	GO:0061134	peptidase regulator activity	3 (8.33%)	39 (0.84%)	0.003278	**0.011920**
34	GO:0022857	transmembrane transporter activity	6 (16.67%)	196 (4.24%)	0.003629	**0.012373**
35	GO:0008094	DNA-dependent ATPase activity	2 (5.56%)	12 (0.26%)	0.003712	**0.012373**
36	GO:0050780	dopamine receptor binding	2 (5.56%)	12 (0.26%)	0.003712	**0.012373**
37	GO:0046872	metal ion binding	13 (36.11%)	774 (16.76%)	0.003942	**0.012785**
38	GO:0016462	pyrophosphatase activity	9 (25%)	423 (9.16%)	0.004156	**0.013124**
39	GO:0016818	hydrolase activity, acting on acid anhydrides, in phosphorus-containing anhydrides	9 (25%)	427 (9.24%)	0.004427	**0.013621**
40	GO:0016817	hydrolase activity, acting on acid anhydrides	9 (25%)	429 (9.29%)	0.004568	**0.013703**
41	GO:0055102	lipase inhibitor activity	2 (5.56%)	15 (0.32%)	0.005819	**0.017031**
42	GO:0043167	ion binding	17 (47.22%)	1302 (28.19%)	0.011415	**0.032616**
43	GO:0004601	peroxidase activity	2 (5.56%)	22 (0.48%)	0.012371	**0.034525**
44	GO:0016787	hydrolase activity	14 (38.89%)	995 (21.54%)	0.013318	**0.035957**
45	GO:0016684	oxidoreductase activity, acting on	2 (5.56%)	23 (0.5%)	0.013484	**0.035957**

		peroxide as acceptor				
46	GO:0043168	anion binding	6 (16.67%)	260 (5.63%)	0.014036	**0.036615**
47	GO:0043169	cation binding	15 (41.67%)	1118 (24.2%)	0.015299	**0.039060**

C. Cellular Component

#	GO ID	Description	Freshwater vs Salinity-Alkalinity UP (41)	All (4741)	*p-value*	*p-adjust*
1	GO:0005886	plasma membrane	13 (31.71%)	462 (9.74%)	0.000082	**0.006010**
2	GO:0071944	cell periphery	13 (31.71%)	488 (10.29%)	0.000145	**0.006010**
3	GO:0098794	postsynapse	4 (9.76%)	36 (0.76%)	0.000232	**0.006010**
4	GO:0005576	extracellular region	12 (29.27%)	445 (9.39%)	0.000253	**0.006010**
5	GO:0048770	pigment granule	5 (12.2%)	74 (1.56%)	0.000391	**0.007374**
6	GO:0044291	cell-cell contact zone	4 (9.76%)	43 (0.91%)	0.000466	**0.007374**
7	GO:0044421	extracellular region part	11 (26.83%)	427 (9.01%)	0.000727	**0.009865**
8	GO:0043197	dendritic spine	2 (4.88%)	6 (0.13%)	0.001071	**0.012717**
9	GO:0044456	synapse part	5 (12.2%)	98 (2.07%)	0.001415	**0.014941**
10	GO:0014069	postsynaptic density	3 (7.32%)	31 (0.65%)	0.002281	**0.018060**
11	GO:0060076	excitatory synapse	3 (7.32%)	31 (0.65%)	0.002281	**0.018060**
12	GO:0099572	postsynaptic specialization	3 (7.32%)	31 (0.65%)	0.002281	**0.018060**
13	GO:0031988	membrane-bounded vesicle	24 (58.54%)	1701 (35.88%)	0.002476	**0.018097**
14	GO:0031982	vesicle	24 (58.54%)	1739 (36.68%)	0.003473	**0.023566**
15	GO:0045202	synapse	5 (12.2%)	132 (2.78%)	0.005197	**0.032913**
16	GO:0016023	cytoplasmic, membrane-bounded vesicle	8 (19.51%)	324 (6.83%)	0.005697	**0.033824**
17	GO:0031410	cytoplasmic vesicle	8 (19.51%)	334 (7.04%)	0.006832	**0.038181**
18	GO:0044459	plasma membrane part	8 (19.51%)	340 (7.17%)	0.007593	**0.040072**
19	GO:0005764	lysosome	2 (4.88%)	23 (0.49%)	0.016459	0.080787
20	GO:0001674	female germ cell nucleus	1 (2.44%)	2 (0.04%)	0.017223	0.080787
21	GO:0030425	dendrite	2 (4.88%)	24 (0.51%)	0.017858	0.080787
22	GO:0036477	somatodendritic compartment	2 (4.88%)	29 (0.61%)	0.025566	0.101830
23	GO:0030175	filopodium	1 (2.44%)	3 (0.06%)	0.025726	0.101830
24	GO:0031074	nucleocytoplasmic shuttling complex	1 (2.44%)	3 (0.06%)	0.025726	0.101830
25	GO:0005911	cell-cell junction	4 (9.76%)	140 (2.95%)	0.031685	0.120403
26	GO:0016021	integral component of membrane	6 (14.63%)	296 (6.24%)	0.039905	0.145807
27	GO:0031224	intrinsic component of membrane	12 (29.27%)	845 (17.82%)	0.048968	0.166430

Table S5. Pathway Enrichment of differentially expressed proteins under stresses

A. Saline-alkaline stress

Pathway	Candidate proteins with pathway annotation (55)	All proteins with pathway annotation (3593)	*P-value*	*q-value*	Pathway ID
Mineral absorption	7 (12.73%)	28 (0.78%)	0.000000	**0.000019**	ko04978
Thyroid hormone synthesis	7 (12.73%)	40 (1.11%)	0.000002	**0.000107**	ko04918
Carbohydrate digestion and absorption	6 (10.91%)	27 (0.75%)	0.000002	**0.000107**	ko04973
Aldosterone-regulated sodium reabsorption	6 (10.91%)	29 (0.81%)	0.000004	**0.000107**	ko04960
Proximal tubule bicarbonate reclamation	6 (10.91%)	29 (0.81%)	0.000004	**0.000107**	ko04964
Pancreatic secretion	7 (12.73%)	55 (1.53%)	0.000015	**0.000297**	ko04972
Salivary secretion	6 (10.91%)	37 (1.03%)	0.000016	**0.000297**	ko04970
Bile secretion	6 (10.91%)	37 (1.03%)	0.000016	**0.000297**	ko04976
Insulin secretion	6 (10.91%)	38 (1.06%)	0.000018	**0.000310**	ko04911
Protein digestion and absorption	7 (12.73%)	59 (1.64%)	0.000025	**0.000371**	ko04974
Endocrine and other factor-regulated calcium reabsorption	6 (10.91%)	43 (1.2%)	0.000038	**0.000527**	ko04961
Aldosterone synthesis and secretion	6 (10.91%)	46 (1.28%)	0.000057	**0.000717**	ko04925
Gastric acid secretion	6 (10.91%)	50 (1.39%)	0.000092	**0.001071**	ko04971
Staphylococcus aureus infection	6 (10.91%)	59 (1.64%)	0.000235	**0.002537**	ko05150
Transcriptional misregulation in cancers	6 (10.91%)	75 (2.09%)	0.000871	**0.008766**	ko05202
Cardiac muscle contraction	6 (10.91%)	84 (2.34%)	0.001582	**0.014925**	ko04260
Legionellosis	5 (9.09%)	63 (1.75%)	0.002501	**0.022219**	ko05134
Arachidonic acid metabolism	4 (7.27%)	39 (1.09%)	0.002717	**0.022387**	ko00590
cAMP signaling pathway	6 (10.91%)	94 (2.62%)	0.002817	**0.022387**	ko04024
cGMP - PKG signaling pathway	6 (10.91%)	99 (2.76%)	0.003656	**0.027601**	ko04022
Complement and coagulation cascades	4 (7.27%)	43 (1.2%)	0.003896	**0.028015**	ko04610
Thyroid hormone signaling pathway	6 (10.91%)	103 (2.87%)	0.004451	**0.030550**	ko04919
Adrenergic signaling in cardiomyocytes	6 (10.91%)	105 (2.92%)	0.004894	**0.032128**	ko04261

B. Saline stress

Pathway	Candidate proteins with pathway annotation (65)	All proteins with pathway annotation (3593)	*P-value*	*q-value*	Pathway ID
Salivary secretion	7 (10.77%)	37 (1.03%)	0.000003	**0.000129**	ko04970
Bile secretion	7 (10.77%)	37 (1.03%)	0.000003	**0.000129**	ko04976
Insulin secretion	7 (10.77%)	38 (1.06%)	0.000004	**0.000129**	ko04911

Thyroid hormone synthesis	7 (10.77%)	40 (1.11%)	0.000005	**0.000129**	ko04918
Carbohydrate digestion and absorption	6 (9.23%)	27 (0.75%)	0.000006	**0.000129**	ko04973
Mineral absorption	6 (9.23%)	28 (0.78%)	0.000008	**0.000129**	ko04978
Protein digestion and absorption	8 (12.31%)	59 (1.64%)	0.000008	**0.000129**	ko04974
Aldosterone-regulated sodium reabsorption	6 (9.23%)	29 (0.81%)	0.000010	**0.000129**	ko04960
Proximal tubule bicarbonate reclamation	6 (9.23%)	29 (0.81%)	0.000010	**0.000129**	ko04964
Pancreatic secretion	7 (10.77%)	55 (1.53%)	0.000047	**0.000570**	ko04972
Endocrine and other factor-regulated calcium reabsorption	6 (9.23%)	43 (1.2%)	0.000100	**0.001111**	ko04961
Aldosterone synthesis and secretion	6 (9.23%)	46 (1.28%)	0.000147	**0.001499**	ko04925
Gastric acid secretion	6 (9.23%)	50 (1.39%)	0.000236	**0.002100**	ko04971
Lysosome	8 (12.31%)	94 (2.62%)	0.000241	**0.002100**	ko04142
Cardiac muscle contraction	7 (10.77%)	84 (2.34%)	0.000690	**0.005613**	ko04260
Thyroid hormone signaling pathway	7 (10.77%)	103 (2.87%)	0.002303	**0.017564**	ko04919
Staphylococcus aureus infection	5 (7.69%)	59 (1.64%)	0.003910	**0.028061**	ko05150
Cholesterol metabolism	3 (4.62%)	20 (0.56%)	0.005171	**0.035050**	ko04979
cAMP signaling pathway	6 (9.23%)	94 (2.62%)	0.006509	**0.041798**	ko04024

C. Alkaline stress

**Pathway**	**Candidate proteins with pathway annotation (16)**	**All proteins with pathway annotation (3593)**	***P-value***	***q-value***	**Pathway ID**
Protein digestion and absorption	7 (43.75%)	59 (1.64%)	0.000000	**0.000000**	ko04974
Pancreatic secretion	4 (25%)	55 (1.53%)	0.000078	**0.002498**	ko04972
Thyroid hormone synthesis	3 (18.75%)	40 (1.11%)	0.000648	**0.013817**	ko04918
Other glycan degradation	2 (12.5%)	15 (0.42%)	0.001888	**0.025902**	ko00511
ECM-receptor interaction	3 (18.75%)	59 (1.64%)	0.002024	**0.025902**	ko04512
C5-Branched dibasic acid metabolism	1 (6.25%)	1 (0.03%)	0.004453	**0.044213**	ko00660
Carbohydrate digestion and absorption	2 (12.5%)	27 (0.75%)	0.006117	**0.044213**	ko04973
Mineral absorption	2 (12.5%)	28 (0.78%)	0.006570	**0.044213**	ko04978
Aldosterone-regulated sodium reabsorption	2 (12.5%)	29 (0.81%)	0.007038	**0.044213**	ko04960
Proximal tubule bicarbonate reclamation	2 (12.5%)	29 (0.81%)	0.007038	**0.044213**	ko04964
PI3K-Akt signaling pathway	4 (25%)	185 (5.15%)	0.007599	**0.044213**	ko04151

Table S6. GO and pathway enrichment of hub genes under salinity stress

A. Biological Process

**#**	**GO ID**	**Description**	**Saline hub genes (15)**	**All (4696)**	***p-value***	***p-adjust***
1	GO:0022617	extracellular matrix disassembly	2 (13.33%)	5 (0.11%)	0.000095	**0.032489**
2	GO:0009268	response to pH	2 (13.33%)	11 (0.23%)	0.000515	0.088361
3	GO:0001974	blood vessel remodeling	1 (6.67%)	1 (0.02%)	0.003194	0.218796
4	GO:0000460	maturation of 5.8S rRNA	1 (6.67%)	2 (0.04%)	0.006379	0.218796
5	GO:0000470	maturation of LSU-rRNA	1 (6.67%)	2 (0.04%)	0.006379	0.218796
6	GO:0007622	rhythmic behavior	1 (6.67%)	2 (0.04%)	0.006379	0.218796
7	GO:0022410	circadian sleep/wake cycle process	1 (6.67%)	2 (0.04%)	0.006379	0.218796
8	GO:0042273	ribosomal large subunit biogenesis	1 (6.67%)	2 (0.04%)	0.006379	0.218796
9	GO:0042745	circadian sleep/wake cycle	1 (6.67%)	2 (0.04%)	0.006379	0.218796
10	GO:0048512	circadian behavior	1 (6.67%)	2 (0.04%)	0.006379	0.218796
11	GO:0010715	regulation of extracellular matrix disassembly	1 (6.67%)	3 (0.06%)	0.009554	0.273087
12	GO:1903053	regulation of extracellular matrix organization	1 (6.67%)	3 (0.06%)	0.009554	0.273087
13	GO:0022613	ribonucleoprotein complex biogenesis	3 (20%)	150 (3.19%)	0.010959	0.289144
14	GO:0016072	rRNA metabolic process	2 (13.33%)	53 (1.13%)	0.011947	0.290859
15	GO:0051931	regulation of sensory perception	1 (6.67%)	4 (0.09%)	0.012720	0.290859
16	GO:0009719	response to endogenous stimulus	5 (33.33%)	493 (10.5%)	0.015360	0.307270
17	GO:0031644	regulation of neurological system process	1 (6.67%)	5 (0.11%)	0.015876	0.307270
18	GO:0030198	extracellular matrix organization	2 (13.33%)	62 (1.32%)	0.016125	0.307270
19	GO:0035272	exocrine system development	1 (6.67%)	7 (0.15%)	0.022160	0.382124

20	GO:0009725	response to hormone	4 (26.67%)	355 (7.56%)	0.022462	0.382124
21	GO:0048545	response to steroid hormone	3 (20%)	199 (4.24%)	0.023395	0.382124
22	GO:0000302	response to reactive oxygen species	2 (13.33%)	79 (1.68%)	0.025464	0.397008
23	GO:0030218	erythrocyte differentiation	1 (6.67%)	10 (0.21%)	0.031517	0.450425
24	GO:0034101	erythrocyte homeostasis	1 (6.67%)	10 (0.21%)	0.031517	0.450425
25	GO:0045995	regulation of embryonic development	1 (6.67%)	11 (0.23%)	0.034617	0.474942
26	GO:0015669	gas transport	1 (6.67%)	12 (0.26%)	0.037708	0.480383
27	GO:0035264	multicellular organism growth	1 (6.67%)	13 (0.28%)	0.040789	0.480383
28	GO:0040014	regulation of multicellular organism growth	1 (6.67%)	13 (0.28%)	0.040789	0.480383
29	GO:0032870	cellular response to hormone stimulus	2 (13.33%)	108 (2.3%)	0.045280	0.480383
30	GO:0006364	rRNA processing	1 (6.67%)	16 (0.34%)	0.049979	0.480383
31	GO:0045444	fat cell differentiation	1 (6.67%)	16 (0.34%)	0.049979	0.480383

B. Molecular Function

#	GO ID	Description	Saline hub genes (16)	All (4619)	*p-value*	*p-adjust*
1	GO:0070011	peptidase activity, acting on L-amino acid peptides	3 (18.75%)	185 (4.01%)	0.024086	0.397748
2	GO:0008233	peptidase activity	3 (18.75%)	189 (4.09%)	0.025475	0.397748
3	GO:0005501	retinoid binding	1 (6.25%)	10 (0.22%)	0.034137	0.397748
4	GO:0019840	isoprenoid binding	1 (6.25%)	10 (0.22%)	0.034137	0.397748
5	GO:0004175	endopeptidase activity	2 (12.5%)	105 (2.27%)	0.049928	0.397748

C. Cellular Component

#	GO ID	Description	STOP5CombinedHUBGENEs (16)	All (4741)	*p-value*	*p-adjust*
1	GO:0000323	lytic vacuole	4 (25%)	114 (2.4%)	0.000462	**0.023081**
2	GO:0005773	vacuole	4 (25%)	195 (4.11%)	0.003421	0.085525

3	GO:0005576	extracellular region	5 (31.25%)	445 (9.39%)	0.012990	0.167941
4	GO:0030684	preribosome	1 (6.25%)	4 (0.08%)	0.013435	0.167941
5	GO:0005770	late endosome	1 (6.25%)	11 (0.23%)	0.036541	0.365408
6	GO:0044421	extracellular region part	4 (25%)	427 (9.01%)	0.049380	0.411503

D. pathway enrichment

#	Pathway	Candidate genes with pathway annotation (6)	All genes with pathway annotation (3593)	Pvalue	Qvalue	Pathway ID
1	Lysosome	3 (50%)	94 (2.62%)	0.000328	**0.004587**	ko04142
2	Antigen processing and presentation	2 (33.33%)	84 (2.34%)	0.007622	0.053355	ko04612
3	Apoptosis	2 (33.33%)	112 (3.12%)	0.013308	0.062106	ko04210
4	Prion diseases	1 (16.67%)	20 (0.56%)	0.032960	0.115358	ko05020
